# Peripheral B‐cell dysregulation is associated with relapse after long‐term quiescence in patients with multiple sclerosis

**DOI:** 10.1111/imcb.12552

**Published:** 2022-05-12

**Authors:** Felix Marsh‐Wakefield, Pierre Juillard, Thomas M Ashhurst, Annette Juillard, Diana Shinko, Givanna H Putri, Mark N Read, Helen M McGuire, Scott N Byrne, Simon Hawke, Georges E Grau

**Affiliations:** ^1^ Vascular Immunology Unit, School of Medical Sciences, Faculty of Medicine and Health The University of Sydney Sydney NSW Australia; ^2^ Liver Injury and Cancer Program Centenary Institute Sydney NSW Australia; ^3^ Human Cancer and Viral Immunology Laboratory The University of Sydney Sydney NSW Australia; ^4^ Sydney Cytometry Core Research Facility The University of Sydney Sydney NSW Australia; ^5^ School of Medical Sciences, Faculty of Medicine and Health The University of Sydney Sydney NSW Australia; ^6^ Ramaciotti Facility for Human Systems Biology The University of Sydney Sydney NSW Australia; ^7^ School of Computer Science The University of Sydney Sydney NSW Australia; ^8^ Translational Immunology Group, School of Medical Sciences, Faculty of Medicine and Health The University of Sydney Sydney NSW Australia; ^9^ Centre for Immunology and Allergy Research The Westmead Institute for Medical Research Westmead NSW Australia; ^10^ Central West Neurology and Neurosurgery Orange NSW Australia

**Keywords:** Alemtuzumab, B cells, mass cytometry, multiple sclerosis

## Abstract

B cells play a major role in multiple sclerosis (MS), with many successful therapeutics capable of removing them from circulation. One such therapy, alemtuzumab, is thought to reset the immune system without the need for ongoing therapy in a proportion of patients. The exact cells contributing to disease pathogenesis and quiescence remain to be identified. We utilized mass cytometry to analyze B cells from the blood of patients with relapse‐remitting MS (RRMS) before and after alemtuzumab treatment, and during relapse. A complementary RRMS cohort was analyzed by single‐cell RNA sequencing. The R package “Spectre” was used to analyze these data, incorporating FlowSOM clustering, sparse partial least squares‐discriminant analysis and permutational multivariate analysis of variance. Immunoglobulin (Ig)A^+^ and IgG_1_^+^ B‐cell numbers were altered, including higher IgG_1_^+^ B cells during relapse. B‐cell linker protein (BLNK), CD40 and CD210 expression by B cells was lower in patients with RRMS compared with non‐MS controls, with similar results at the transcriptomic level. Finally, alemtuzumab restored BLNK, CD40 and CD210 expression by IgA^+^ and IgG_1_^+^ B cells, which was altered again during relapse. These data suggest that impairment of IgA^+^ and IgG_1_^+^ B cells may contribute to MS pathogenesis, which can be restored by alemtuzumab.

## Graphical abstract

B cells play an important role in the pathogenesis of multiple sclerosis (MS). We undertook a high‐dimensional analysis of B cell subsets in MS patients after treatment with alemtuzumab. Our investigation found IgA^+^ and IgG1^+^ B cells were dysregulated in relapsing MS patients.

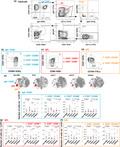

## INTRODUCTION

Alemtuzumab is a humanized monoclonal antibody targeting CD52‐expressing cells, which are primarily expressed by T and B cells. Alemtuzumab, which is used to treat people with multiple sclerosis (MS), leads to the depletion of T‐cell subsets as well as unswitched memory, switched memory and double‐negative B cells, with an overall increase in total B cells accompanied by a relative increase in the proportion of transitional and naïve B‐cell subsets during repopulation.^1^, ^2^, ^3^, ^4^, ^5^, ^6^, ^7^ This dramatic change in B‐cell subsets is hypothesized to explain the success of alemtuzumab as an MS disease‐modifying therapy (DMT).^8^ T‐cell repopulation is much slower and can take years.^5^, ^7^ A remarkable feature of this DMT is that patients with MS treated with alemtuzumab can display long‐lasting protection from disease progression that lasts years without the need for continuous treatment.^9^ However, a subset of patients with MS treated with alemtuzumab undergo inexplicable relapse after the second course. Understanding the reasons for this relapse will allow us to better predict treatment failure and identify the specific immune cells responsible.

One explanation for the relapse occurring in some patients with MS treated with alemtuzumab is a defect in the repopulating B‐cell compartment. To investigate this, we have used mass cytometry combined with FlowSOM clustering^10^ and sparse partial least squares‐discriminant analysis (sPLS‐DA) to identify and analyze 33 distinct B‐cell subsets in patients with MS before and after alemtuzumab treatment, including long‐term stable patients with MS as well as those who have relapsed. Our in‐depth immunophenotyping of B cells provided new insights into the effect of alemtuzumab on B‐cell subsets. A complementary MS cohort was used to interrogate the transcriptome of B cells by single‐cell RNA sequencing and support the proteomic data generated by mass cytometry. The decreased level of B‐cell linker protein (BLNK), CD40 and CD210 expression in untreated patients with MS likely influences the function of various B‐cell subsets, including potentially protective roles of immunoglobulin (Ig)A^+^CD20^+^ B cells and detrimental IgG_1_^+^ B cells. The data shown here highlight the complexity of MS, and the contribution of many B‐cell subsets that could influence disease outcome. Future therapeutics may benefit from inhibiting the activation of IgG_1_^+^ B cells while promoting the regulatory functions of IgA^+^ B cells.

## RESULTS

### Clustered B‐cell repertoire differs between disease and treatment groups

**Figure 1:**
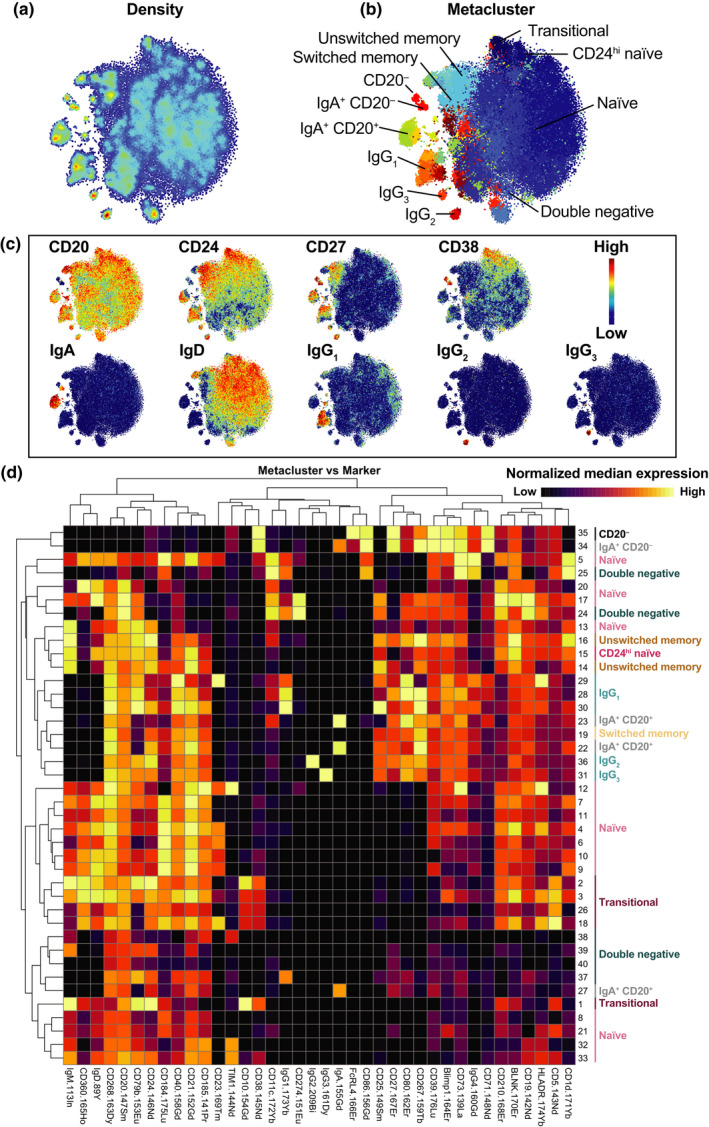
Clustered B‐cell repertoire differs between disease and treatment groups. FlowSOM clustering was first done on B cells from each patient. A total of 40 metaclusters were generated. **(a–c)** Data were then downsampled evenly across groups to 6000 cells to create FIt‐SNE plots. Colors represent labeled parameter (density, metacluster, CD20, CD24, CD27, CD38, IgA, IgD, IgG_1_, IgG_2_, IgG_3_). **(d)** A heatmap was generated to visualize the relative marker expression (columns) across metaclusters (rows). Each marker was rescaled by minimum/maximum, to compare high (yellow) and low (black) marker expression. Median signal intensity for each marker was calculated for each metacluster. Markers (columns) were rescaled by minimum/maximum. Each metacluster was assigned to one of the conventional B‐cell subsets (transitional, naïve, CD24^hi^ naïve, unswitched memory, switched memory, double negative, IgG_3_, IgG_1_, IgG_2_, IgA^+^CD20^+^, IgA^+^CD20^−^, CD20^−^ B cells). FIt‐SNE, fast interpolation‐based t‐distributed stochastic neighbor embedding; Ig, immunoglobulin.

FlowSOM clustering was first performed to examine the overall B‐cell compartment. Fast interpolation‐based t‐distributed stochastic neighbor embedding (FIt‐SNE) plots (Figure 1a–c) and heatmaps (Figure 1d) were used to visualize the success of clustering to identify conventional B‐cell subsets (transitional, naïve, CD24^hi^ naïve, unswitched memory, switched memory, double‐negative, IgG_3_, IgG_1_, IgG_2_, IgG_4_, IgA^+^CD20^+^, IgA^+^CD20^−^, CD20^−^ B cells).

**Figure 2:**
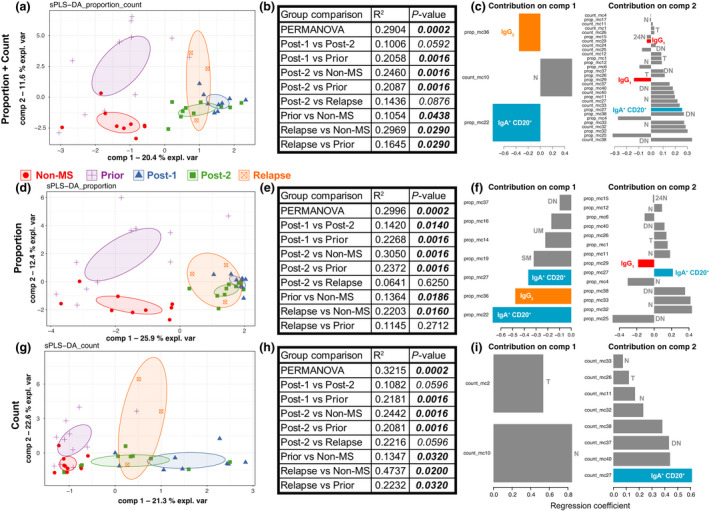
Overall B‐cell compartment differs between groups. FlowSOM clustering was done to first identify 40 metaclusters. The levels of each metacluster were calculated as proportion of B cells or counts for each patient. sPLS‐DA plots were generated to visualize differences between groups, using **(a)** proportion + count, **(d)** proportion or **(g)** count data. Each symbol represents an individual patient. Symbol and colors represent the group each patient belongs to. Ellipses are 95% confidence intervals. **(b**, **e**, **h)** A PERMANOVA was performed using the metaclusters identified by sPLS‐DA. Results for each comparison are shown in the relevant table. **(c**, **f**, **i)** The metaclusters used in the first two components of the sPLS‐DA are shown. The conventional B‐cell subset each metacluster belongs to is annotated. *24N*, CD24^hi^ naïve; *DN*, double negative; Ig, immunoglobulin; MS, multiple sclerosis; *N*, naïve; *T*, transitional; PERMANOVA, permutational multivariate analysis of variance; *SM*, switched memory; sPLS‐DA, sparse partial least squares‐discriminant analysis; *UM*, unswitched memory.

To determine whether the overall B‐cell repertoire differed between groups (and to identify metaclusters that contributed to these differences), an sPLS‐DA was performed. The quantity of each metacluster was calculated as a proportion of B cells and the absolute cell count for each patient. The sPLS‐DA plots suggested there was a difference between groups (Figure 2a, d, g), which was confirmed by a permutational multivariate analysis of variance (PERMANOVA) using the selected metaclusters highlighted by the sPLS‐DA. Of particular note, there was a clear difference between untreated patients/controls compared with patients with MS treated with alemtuzumab. Statistically significant differences were found between *non‐MS* and *prior*, *non‐MS* and *post‐2*, *non‐MS* and *relapse*, *prior* and *post‐1*, *prior* and *post‐2*, *prior* and *relapse* (Figure 2b, e, h). The metaclusters used in the first two components are shown (Figure 2c, f, i). Each row represents a different metacluster, with annotations indicating the conventional B‐cell subset each metacluster belongs to. Metaclusters are ranked based on their contribution to each component, with the largest contributor at the bottom. In combined proportion + count (Figure 2a–c), both proportion and count data from multiple B‐cell subsets contributed to differences between the groups. This included contributions from subsets previously reported to be involved in MS pathogenesis and affected by alemtuzumab treatment (transitional, naïve, double‐negative, unswitched memory and switched memory B cells) as well as novel subsets, particularly IgA^+^CD20^+^, IgG_1_^+^ and IgG_2_^+^ B cells. Furthermore, IgG_2_^+^ B cells contributed to component 1, which separates *non‐MS* controls and *prior* patients from treated patients with MS. By contrast, IgG_1_^+^ B cells contributed to component 2, which differentiates *non‐MS* controls from *prior* patients. IgA^+^CD20^+^ B cells contributed to both components. In component 1 (*x*‐axis), there is clear separation between untreated (*non‐MS*, *prior*) and treated (*post‐1*, *post‐2*, *relapse*). In component 2 (*y*‐axis), *non‐MS* and *prior* are separated. Furthermore, two of the three patients with *relapse* begin to shift up toward *prior*, away from *non‐MS*. Although a small *n*‐value, this may suggest the B‐cell repertoire in patients with *relapse* is more similar to *prior* patients than *non‐MS* controls.

### IgA ^+^ CD20 ^+^, IgG1 ^+^ and IgG2 ^+^ B cells are affected by alemtuzumab treatment

**Figure 3:**
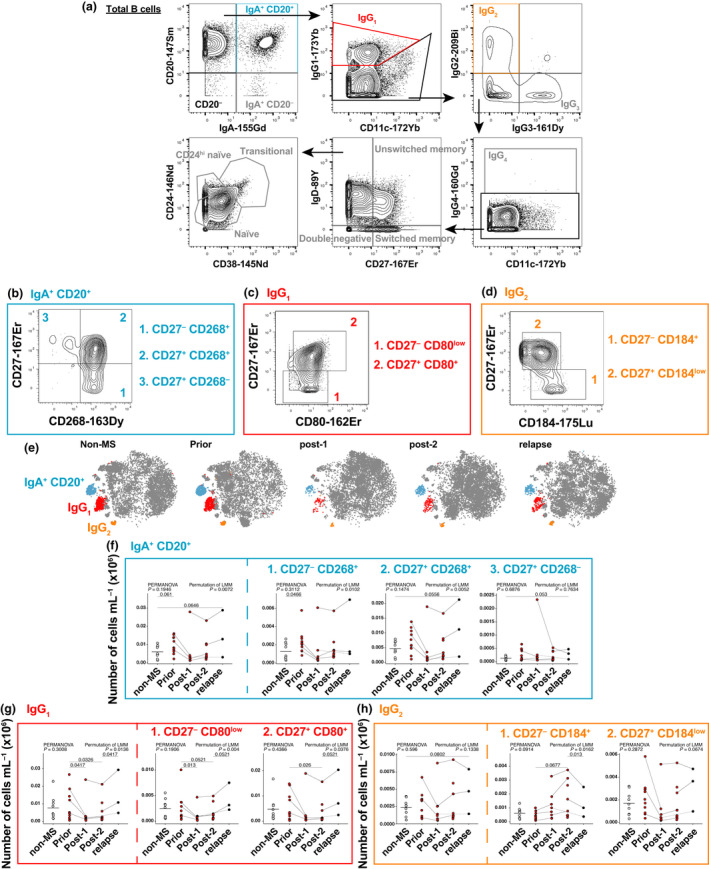
IgA^+^CD20^+^, IgG_1_^+^ and IgG_2_^+^ B cells are affected by alemtuzumab treatment and associated with relapse. **(a)** Manual gating of conventional B‐cell subsets. CD27, CD80, CD184 and CD185 were used to further differentiate IgA^+^CD20^+^, IgG_1_^+^ and IgG_2_^+^ B‐cell subsets. Manual gating strategy of more defined **(b)** IgA^+^CD20^+^, **(c)** IgG_1_^+^ and **(d)** IgG_2_^+^ B‐cell subsets. Numbers represent least to most developed B‐cell subset within each subset. **(e)** FIt‐SNE plots represent B‐cell repertoire between groups. Blue cells are IgA^+^CD20^+^ B cells, red cells are IgG_1_^+^ B cells, and orange cells are IgG_2_^+^ B cells. All other B‐cell subsets are dark gray. Counts of **(f)** IgA^+^CD20^+^, **(g)** IgG_1_^+^ and **(h)** IgG_2_^+^ B cells (and their respective subsets) are shown as scatter plots. Solid lines signify data are available for adjacent timepoints, while dotted lines indicate patients with nonadjacent timepoints. For comparisons of B‐cell subset levels between all five groups [*non‐MS* controls (*n* = 9), patients with untreated MS (*prior*, *n* = 11) and patients with MS *post‐1* (up to 12 months after alemtuzumab dose, *n* = 8), *post‐2* (greater than 12 months, *n* = 10) alemtuzumab and *relapse* (*n* = 3)], a PERMANOVA was performed followed by pairwise comparisons with Holm’s correction. *Prior*, *post‐2* and *relapse* groups were compared with *non‐MS* controls (for three comparisons). An LMM was calculated when comparing between patients with MS before and after treatment. A total of 4999 permutations were then run to calculate *P*‐values. Five multiple comparisons were made (*prior* to *post‐1*, *post‐2* and *relapse*; and *post‐*1 to *post‐2* and *post‐2* to *relapse*) using a further 4999 permutations with Holm’s correction. The mean is shown in *non‐MS* controls, *P*‐values < 0.1 are shown. Ig, immunoglobulin; LMM, linear mixed‐effects model; MS, multiple sclerosis; PERMANOVA, permutational multivariate analysis of variance.

To validate the clustering results of IgA^+^CD20^+^, IgG_1_^+^ and IgG_2_^+^ B cells in MS pathogenesis and response to alemtuzumab treatment, manual gating was performed to identify conventional B‐cell subsets (Figure 3a, Supplementary figure 1). The results from clustering guided further subset differentiation, whereby CD27 together with CD80, CD184 or CD268 was used to differentiate IgG_1_^+^, IgG_2_^+^ and IgA^+^CD20^+^ B‐cell subsets, respectively (Figure 3b–d). A similar approach was used to differentiate other conventional B‐cell subsets (Supplementary figure 1c n). FIt‐SNE plots visualize differences between groups (Figure 3e). IgA^+^CD20^+^CD27^−^CD268^+^ B cells were significantly higher in patients with MS *prior* to alemtuzumab compared with *non‐MS* controls (Figure 3f and Supplementary figure 2 m for proportions). While the overall number and proportion of B cells rises after alemtuzumab treatment (Supplementary figure 2b), total IgG_1_^+^ as well as IgG_1_^+^CD27^−^CD80^low^ and IgG_1_^+^CD27^+^CD80^+^ B‐cell subsets all significantly decreased after alemtuzumab treatment (Figure 3g and Supplementary figure 2j for proportions). Strikingly, despite the low number of relapses in this cohort (*n* = 3), MS activity was associated with a statistically significant rise in IgG1^+^ B cells. By contrast, IgG_2_^+^CD27^−^CD184^+^ B cells decreased during MS *relapse* (Figure 3h). These differences appear independent of MS activity and previous treatments (Supplementary figure 3).

### BLNK and CD210 expression on B cells are significantly lower in patients with MS

**Figure 4:**
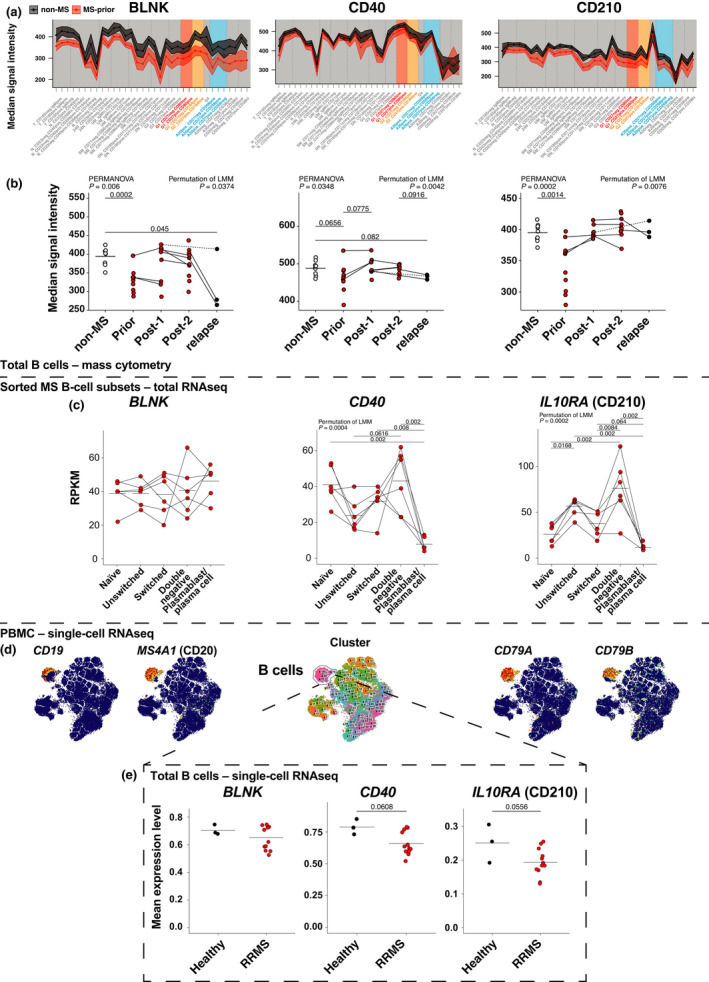
B‐cell protein and transcript expression of BLNK, CD40 and CD210 are decreased in patients with untreated MS, but are restored after alemtuzumab treatment. **(a)** Median signal intensity of BLNK, CD40 and CD210 were calculated across B‐cell subsets of *non‐MS* (black) and *prior* (red) patients. Mean and 95% confidence intervals are shown. **(b)** Median signal intensity of total B‐cell expression of BLNK, CD40 and CD210 is shown across groups. Solid lines signify data are available for adjacent timepoints, while dotted lines indicate patients with nonadjacent timepoints. For comparisons of B‐cell subset levels between all five groups [*non‐MS* controls (*n* = 9), patients with untreated MS (*prior*, *n* = 11) and patients with MS *post‐1* (up to 12 months after alemtuzumab dose, *n* = 9), *post‐2* (greater than 12 months, *n* = 10) alemtuzumab and *relapse* (n = 3)], a PERMANOVA was performed followed by pairwise comparisons with Holm’s correction. *Prior*, *post‐2* and *relapse* groups were compared with *non‐MS* controls (for three comparisons). A LMM was calculated when comparing between patients with MS before and after treatment. A total of 4999 permutations were then run to calculate *P*‐values. Five multiple comparisons were made (*prior* to *post‐1*, *post‐2* and *relapse*; and *post‐*1 to *post‐2* and *post‐2* to *relapse*) using a further 4999 permutations with Holm’s correction. The mean is shown in *non‐MS* controls, *P*‐values < 0.1 are shown. **(c)** Five B‐cell subsets, naïve (IgD^+^CD27^−^), double negative (IgD CD27^−^), unswitched memory (IgD^+^CD27^+^), switched memory (IgD^−^CD27^+^) and plasmablasts/plasma cells (IgD^−^CD27^hi^), were first sorted and then underwent total RNA sequencing and calculated RPKM. The levels of *BLNK*, *CD40* and *IL10RA* (CD210) were calculated. Solid lines signify data are available for adjacent timepoints. A LMM was calculated when comparing between patients with MS before and after treatment. A total of 4999 permutations were then run to calculate *P*‐values. Each group was compared with each other (10 comparisons) using a further 4999 permutations with Holm’s correction. The mean is shown for each subset. **(d)** Single‐cell RNA sequencing was performed on PBMCs from patients with RRMS and healthy controls. Clustering was first done to identify B cells, with FIt‐SNE plots for visualization. **(e)** Transcript levels of *BLNK*, *CD40* and *IL10RA*. Permutation of *t*‐test, the mean is shown. *24 N*, CD24^hi^ B cells; *A20* ^*+*^, IgA^+^CD20^+^ B cells; *A20* ^*−*^, IgA^+^CD20^−^ B cells; *CD20* ^*−*^, CD20^−^ B cells; *DN*, double negative B cells; *G1*, IgG_1_^+^ B cells; *G2*, IgG_2_^+^ B cells; *G3*, IgG_3_^+^ B cells; Ig, immunoglobulin; IL, interleukin; MS, multiple sclerosis; *N*, naïve B cells; PBMCs, peripheral blood‐derived mononuclear cells; PERMANOVA, permutational multivariate analysis of variance; RPKM, reads per kilobase per million; RRMS, relapse‐remitting MS; *SM*, switched memory B cells; *T*, transitional B cells; *UM*, unswitched memory B cells.

**Figure 5:**
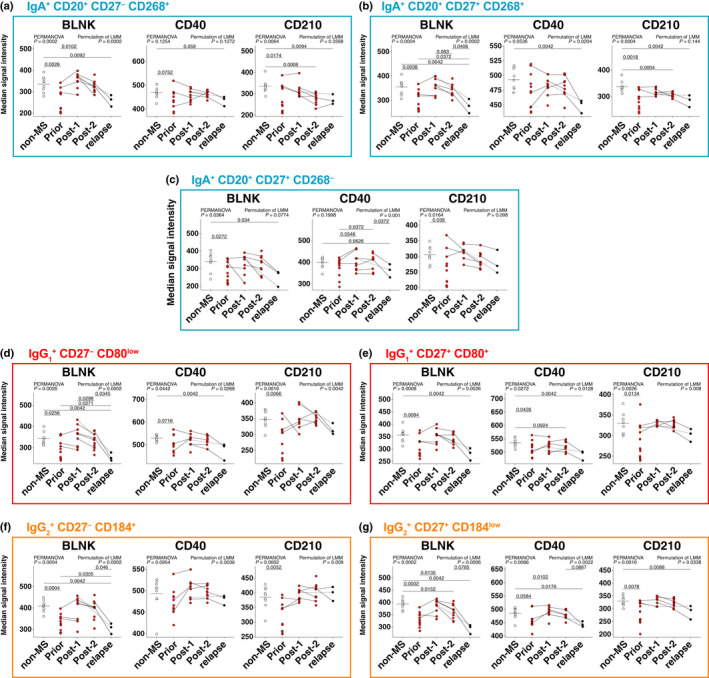
BLNK, CD40 and CD210 levels are altered in IgA^+^CD20^+^, IgG_1_^+^ and IgG_2_^+^ B cells during treatment. The median signal intensity of BLNK, CD40 and CD210 was calculated for **(a–c)** IgA^+^CD20^+^, **(d, e)** IgG_1_^+^ and **(f, g)** IgG_2_^+^ B‐cell subsets. Solid lines signify data are available for adjacent timepoints, while dotted lines indicate patients with nonadjacent timepoints. For comparisons of B‐cell subset levels between all five groups [*non‐MS* controls (*n* = 9), patients with untreated MS (*prior*, *n* = 11) and patients with MS *post‐1* (up to 12 months after alemtuzumab dose, *n* = 9), *post‐2* (greater than 12 months, *n* = 10) alemtuzumab and *relapse* (*n* = 3)], a PERMANOVA was performed (with *P*‐values indicated on all figures) followed by pairwise comparisons with Holm’s correction. *Prior*, *post‐2* and *relapse* groups were compared with *non‐MS* controls (for three comparisons). A LMM was calculated when comparing between patients with MS before and after treatment (with *P*‐values indicated on all figures). A total of 4999 permutations were then run to calculate *P*‐values. Five multiple comparisons were made (*prior* to *post‐1*, *post‐2* and *relapse*; and *post‐*1 to *post‐2* and *post‐2* to *relapse*) using a further 4999 permutations with Holm’s correction. The mean is shown in *non‐MS* controls, *P*‐values < 0.1 are shown. Ig, immunoglobulin; LMM, linear mixed‐effects model; MS, multiple sclerosis; PERMANOVA, permutational multivariate analysis of variance.

We made use of our comprehensive mass cytometry panel to interrogate B‐cell expression of a range of markers that may be associated with MS pathogenesis and/or affected by alemtuzumab treatment (Supplementary figures 4 and 5). We discovered that although BLNK, CD40 and CD210 were constitutively expressed across most subsets (Figure 4a), the overall expression of these markers by B cells was lower in patients with untreated MS compared with *non‐MS* controls (Figure 4b). To confirm our observation in a second cohort and determine whether similar changes occurred at the transcriptomic level, single‐cell and total RNA sequencing data were analyzed on the publicly available data set GSE133028.^11^ Conventional B‐cell subsets constitutively expressed *BLNK*, *CD40* and *IL10RA* (CD210) with changes in expression as the subsets become more developed (Figure 4c), mirroring those which we observed in our cohort (Figure 4a). For single‐cell data, B cells were identified by FlowSOM clustering (Figure 4d), and transcript levels were compared between healthy controls and patients with untreated relapsing–remitting MS (RRMS; Figure 4e). As seen in our mass cytometry cohort (Figure 4b), there was a downward trend of *BLNK*, *CD40* and *IL10RA* (CD210) in patients with RRMS (*n* = 12) compared with healthy controls (*n* = 3). Finally, we confirmed that BLNK and CD210 expressions were significantly lower in our newly identified subsets of interest: IgA^+^CD20^+^ (Figure 5a–c), IgG_1_^+^ (Figure 5d, e) and IgG_2_^+^ (Figure 5f, g) B cells in people with MS. After treatment with alemtuzumab, BLNK and CD210 expressions were restored to healthy control levels in IgA^+^CD20^+^CD27^+^CD268^+^ B cells (Figure 5b), IgG_1_^+^CD27^−^CD80^low^ (Figure 5d) and both IgG_2_^+^ B‐cell subsets (Figure 5f, g). In patients that relapsed, BLNK expression in these subsets was again lower compared with *post‐2*. CD40 expression was lower in patients with *relapse* compared with *non‐MS* controls. Together, these results suggest a functional impairment of these B‐cell subsets may contribute to disease pathogenesis, which can be restored by alemtuzumab treatment.

## DISCUSSION

Recent advances in MS therapeutics suggest that B cells play a prominent role in the pathogenesis of MS, but how targeting B cells suppresses disease activity is not clear. Key findings to emerge from our analyses were the unexpected changes occurring in circulating IgA^+^, IgG1^+^ and IgG2^+^ B cells. Using the power of mass cytometry, we were able to identify seven discrete B‐cell subsets that were affected by MS disease and altered by alemtuzumab therapy based on their surface expression of CD27, CD80, CD184 and CD268. Deeper interrogation of these novel B‐cell subsets revealed changes in the expression of BLNK, CD40 and CD210 during MS pathogenesis and following the administration of a long‐lasting B‐cell‐targeting DMT.

B‐cell‐derived Igs, particularly IgG_1_, have long been associated with autoimmunity,^12^, ^13^ including MS.^14^, ^15^ Serum levels of IgG_1_ are elevated in patients with MS compared with healthy controls,^16^ with alemtuzumab decreasing total serum IgG in patients with MS.^17^ While we did not detect any difference in circulating IgG_1_^+^ B‐cell subsets between *non‐MS* controls and untreated patients with MS, we did observe a significant DMT effect with significantly lower numbers of IgG_1_^+^ B cells following alemtuzumab. Suggestive of a pathogenic role for IgG_1_^+^ B cells was the significant rise we observed for this subset in the three patients who relapsed.

Serum IgG_2_ levels in patients with MS have been found to be either no different compared with healthy controls^16^ or decreased in both patients with MS and clinically isolated syndrome compared with healthy controls.^18^ This latter study is consistent with the changes in IgG_2_^+^CD27^−^CD184^+^ B cells we observed following treatment and during *relapse*. The dichotomy between IgG_1_ and IgG_2_ is well known, as cytokines such as interferon‐γ can inhibit IgG_1_ while promoting IgG_2_ secretion^19^ and may contribute to the differences between IgG_1_^+^ and IgG_2_^+^ B‐cell levels after DMT and during *relapse*.

The role of IgA‐producing B cells in MS pathogenesis is coming under increased scrutiny. In preclinical models, IgA^+^ B cells have been found to play a protective role against experimental autoimmune encephalomyelitis.^20^ Similarly in humans, IgA^+^CD19^+^CD27^+^ B cells produce more interleukin (IL)‐10 compared with IgD^+^CD27^−^, IgM^+^CD27^+^ and IgG^+^CD27^+^ B cells when stimulated with IL‐21 and CD40L.^21^ Therefore, the higher number of IgA^+^CD20^+^ B cells in patients with MS *prior* to treatment with alemtuzumab compared with *non‐MS* controls may reflect an immunological rheostat attempting to contain the autoinflammation underway. Similar results were observed in a recent study.^22^ This hypothesis is supported by the fact that these B‐cell subsets increase upon *relapse*.

BLNK was first identified as a central linker protein that connects the B‐cell receptor‐associated kinases with other signaling pathways.^23^ BLNK plays an important role in B‐cell development.^24^, ^25^ We have discovered that compared with *non‐MS* controls, BLNK protein levels in people with MS are significantly lower in all circulating B cells analyzed, particularly the IgA^+^, IgG1^+^ and IgG2^+^ subsets we have identified to play a novel role in MS pathogenesis and their response to alemtuzumab. Our observation is supported by a recent study showing lower *BLNK* mRNA levels in patients with benign MS compared with healthy controls.^26^ Lower BLNK levels is likely to result in perturbed B‐cell receptor signaling^27^ and/or B‐cell survival^28^ in MS. It may also indicate a defect in immune regulation as mice deficient in BLNK have impaired IL‐10 production from B10 cells and develop more severe experimental autoimmune encephalomyelitis.^29^ These differences in BLNK expression do not appear to be an inherent defect in the B‐cell compartment, as B cells emerging from the bone marrow following the first round of alemtuzumab treatment had BLNK levels that were comparable to *non‐MS* controls. However, lower BLNK expression in B cells does appear to be a harbinger of disease progression and a potential early indicator of MS relapse.

Like BLNK, levels of the IL‐10 receptor (CD210) were lower in almost all B‐cell subsets analyzed from patients with MS compared with *non‐MS* controls. This suggests that circulating B cells in patients with MS are likely to be resistant to the effects of IL‐10 on growth, differentiation^30^ and survival.^31^ CD210 expression has also been associated with regulatory B‐cell activation in both mice^32^ and humans.^33^ Together with signaling through CD40, B cells respond to IL‐10 by altering their production of IgM, IgG_1_ and IgG_3._ ^34^ Indeed, most B cells, including some of those affected by MS disease and altered by alemtuzumab therapy, expressed lower levels of CD40, a well‐known MS risk gene.^35^ Signaling through CD40 and IL‐21 receptors drive B10‐cell development and expansion in mice, which can inhibit established experimental autoimmune encephalomyelitis.^36^ In humans, CD40 activation can induce B‐cell production of IL‐10 and upregulation of CD210.^33^, ^37^ B cells from patients with MS produce less CD40‐induced IL‐10.^38^, ^39^

A limitation within the study was the low number of *relapse* patients (*n* = 3). Although there were significant differences in B‐cell subset levels between groups, more may be observed with a larger patient cohort. Furthermore, full kinetics from individual patients from *prior* to *relapse* were absent because of limits with sample collection. Samples from patients over the full period would be beneficial in future studies. All three *relapse* patients had also previously received other DMTs prior to alemtuzumab, with no samples taken prior, so the effects observed may not be fully dependent on alemtuzumab. Although B cells undoubtedly play a major role in the pathogenesis of MS, other immune cells (such as T cells^5^) would be of interest in future studies.

Our in‐depth immunophenotyping of B cells has provided new insights into the effect of alemtuzumab on B‐cell subsets. The lower level of BLNK, CD40 and CD210 expression in patients with untreated MS likely influences the function of various B‐cell subsets, including IgA^+^CD20^+^ and IgG_2_^+^ B cells in controlling disease. The increase in IgG_1_^+^ B cells during *relapse* likely contributes to disease pathogenesis, possibly because of a breakdown in regulatory mechanisms. The data shown here highlight the complexity of MS, and the contribution of many B‐cell subsets that will influence disease outcome. Future therapeutics may benefit from restoring BLNK and/or CD210 levels in an effort to inhibit the activation of IgG_1_^+^ B cells while promoting the regulatory functions of IgA^+^ B cells.

## METHODS

### Study participants

Ethical consent for the study was obtained from the Research Integrity and Ethics Administration of the University of Sydney (project numbers 2018/708 and 2018/377). MS was defined by McDonald 2017 criteria and disease activity was defined by neurological signs/symptoms and the presence of new T2 or gadolinium‐enhancing magnetic resonance imaging lesions. No patients were on DMTs at the time of blood sampling. All patients had low Expanded Disability Status Scale scores (range 0–2.5). Patient data are shown in Table 1.

**Table 1:** Patient characteristics.

Patient ID	Sex	Age^a^	Disease duration (years)^a^	Months since first alemtuzumab dose				Previous treatment	Months since last treatment prior to alemtuzumab	Active MS^b^
				Prior	Post‐1	Post‐2	Relapse			
MS	74% F	Median = 40.2	Median = 5.9	*n* = 11	*n* = 9	*n* = 10	*n* = 3		Median = 2	
MS01	F	38.8	5.9		24	32		Fingolimod	2	Yes
MS02	F	36	9.3		21	36	39	Azathioprine and intravenous immunoglobulin	1	Yes
MS03	M	43.1	23.3		19		34	Natalizumab	1	Yes
MS04	F	25.1	3.3			31		–/–	–/–	Yes
MS05	F	34.1	11.5			39		Fingolimod	4	Yes
MS06	F	52	24.5			30	41	Natalizumab	1	No
MS07	F	40.2	6.9		11^c^	36		Dimethyl fumarate	3	Yes
MS08	M	33.1	0.3	X	18			–/–	–/–	Yes
MS09	F	46.9	4.3		22	38		Fingolimod	2	Yes
MS10	F	32	2.8	X	11			Fingolimod	6	Yes
MS11	M	46.2	19			39		–/–	–/–	Yes
MS12	M	53.4	0.1	X	20			–/–	–/–	Yes
MS13	F	40.2	3.3			32		Fingolimod	2	Yes (+ clinical)
MS14	M	46.4	8.8		8			Fingolimod	2	Yes
MS15	F	40.2	6			29		Fingolimod	3	No (but clinical)
MS16	F	25.2	0.3	X				Dimethyl fumarate	2	Yes
MS17	F	37.2	17.3	X				–/–	–/–	Yes
MS18	F	41.2	5.8	X				Fingolimod	15	New lesion
MS19	M	36.7	0.4	X				–/–	–/–	Yes
MS20	F	35.8	6.4	X				Copaxone and interferon‐β	> 12	Yes
MS21	F	37.4	2.4	X				Fingolimod	1	No
MS22	F	44.8	0.1	X				–/–	–/–	Yes
MS23	F	45.5	18.2	X				Fingolimod	14	No

Non‐MS	78% F	Median = 39.0
noMS01	F	40.8
noMS02	F	36.8
noMS03	M	41.4
noMS04	F	23.5
noMS05	F	33.1
noMS06	F	54.9
noMS07	F	39
noMS08	F	27.5
noMS09	M	42.3

F, female; M, male; MS, multiple sclerosis; *Post‐1*, 6–12 months after the first or second dose; *Post‐2*, 29–39 months after the first dose; *Prior*, MS before alemtuzumab; *Relapse,* sample of a patient with MS within 1 week of relapse.

^a^ Age/disease duration when first sample was taken.

^b^ Active MS defined as new T2 or T1 Glad‐enhancing lesion in the 6 months prior to starting alemtuzumab.

^c^ No cell count was available for this sample, so was not used in count data.

A total of 23 patients with MS were included in this study. All patients were reviewed clinically every 6 months and had 3‐T magnetic resonance imaging of brain and spinal cord prior to and 6 months after starting treatment and then every 12 months unless new symptoms developed. Blood samples taken from patients with MS prior to alemtuzumab treatment (*prior*, *n* = 11) were treatment naïve (5/11) or free from treatment for at least 1 month prior to alemtuzumab (6/11). The first course of alemtuzumab was given for 5 consecutive days, with a repeated second course 12 months later over 3 days. Post‐alemtuzumab treatment was divided into three groups. *Post‐1* had blood taken up to 12 months after alemtuzumab dose (8–11 months after the first dose *n* = 3; 6–12 months after the second dose *n* = 6). One of these patients did not have the peripheral blood‐derived mononuclear cells (PBMCs) counted, so eight of nine patients were included for cell count data. *Post‐2* had blood taken 29–39 months after first treatment (> 12 months since second course; *n* = 10). *Relapse* included patients that developed new clinical symptoms after two courses of alemtuzumab (with new lesions confirmed by magnetic resonance imaging) and blood was taken within 1 week of symptoms and prior to corticosteroid or additional alemtuzumab treatment (*n* = 3). Age‐ and sex‐matched *non‐MS* controls (*n* = 9) were included in the study, with samples taken from a single timepoint (Table 1).

### Blood sampling

PBMCs were isolated from blood within 0–8 h of collection in EDTA vacuette tubes (Greiner Bio‐One International, Kremsmünster, Austria) using a Ficoll‐Paque PLUS (GE Healthcare, Chicago, IL) density separation gradient. When blood was not processed immediately, samples were stored at room temperature to minimize cell loss.^40^ Although leaving cells at room temperature can alter receptor expression (particularly chemokine receptors),^40^ variance of receptor expression between patients was comparable to internal controls, suggesting that experiment/instrument variability played a larger role than blood processing time (Supplementary figure 5). Cells were counted and blood volume was recorded, such that the concentration of cells in blood could be calculated. Samples were cryopreserved in 5% dimethyl sulfoxide/fetal bovine serum for storage in liquid nitrogen prior to mass cytometry staining.

### Cell staining and analysis by mass cytometry

About 2.5 × 10^6^ cells were resuscitated by thawing in a 37°C water bath and washed in Roswell Park Memorial Institute medium. Individual patient/timepoint samples were first barcoded with anti‐human CD45 (on four different metal isotopes) and purified human FcR‐binding inhibitor (eBioscience Inc., San Diego, CA) for 30 min, such that four independent samples could be combined for further staining as described.^41^ To control for batch variability, PBMCs taken from a single *non‐MS* control (taken from a single timepoint) were included within each batch as an internal control, used across 25 batches (Supplementary figure 5). These controls were analyzed in the same manner to allow comparisons between batches. Samples were combined (for 7.5–10 × 10^6^ cells) and stained with cisplatin (Fluidigm, South San Francisco, CA) for 5 min as a live/dead marker. Cells were stained with antibodies specific for the markers in Supplementary table 1 for 30 min. These antibodies were purchased unlabeled in a carrier‐protein‐free buffer and conjugated with the indicated metal isotope using the X8 Maxpar conjugation kit (Fluidigm) according to the manufacturer’s protocol. The set of antibodies supplied by the Ramaciotti Facility for Human Systems Biology were conjugated, validated and pretitered in per‐test amounts, as indicated in Supplementary table 1. Cells were fixed in 4% paraformaldehyde for 20 min prior to being incubated in Foxp3 permeabilization buffer (eBioscience Inc.) for 15 min. Cells were then stained with an intracellular antibody cocktail (indicated in Supplementary table 1) for 45 min at room temperature (20–24°C). Cells were finally resuspended in DNA intercalator mix [500 nM iridium intercalator (Fluidigm) in 4% paraformaldehyde] for 20 min at room temperature before being left in the fridge until running on a Helios‐upgraded CyTOF 2 mass cytometer (Fluidigm) within 7 days. Cells were washed in Maxpar Cell Acquisition Solution (Fluidigm) before being resuspended in 10% EQ Four Element Calibration Beads (Fluidigm) in Cell Acquisition Solution at a concentration of 0.8 × 10^6^ cells mL^−1^. Data were normalized to EQ beads using CyTOF Software (Fluidigm).

### Mass cytometry data analysis

Samples were initially gated as shown in Supplementary figure 1a, b using FlowJo version 10.4 (Becton Dickinson, Ashland, OR). All analyses were performed on single live CD3^−^CD19^+^CD20^+/−^ B cells.

Clustering was first performed on total B cells using “FlowSOM,”^10^ generating 40 metaclusters. For “FIt‐SNE” dimensionality reduction,^42^ B cells were downsampled such that each group (batch control, *non‐MS*, *prior*, *post‐1*, *post‐2* and *relapse*) contributed 10 000 B cells each for a total of 60 000 B cells, as part of the “Spectre” package^43^ in R.^44^ Both clustering and dimensionality reduction plots were calculated using the following markers: Blimp‐1, BLNK, CD1d, CD5, CD10, CD11c, CD19, CD20, CD21, CD23, CD24, CD25, CD27, CD38, CD39, CD40, CD71, CD73, CD79b, CD80, CD86, CD138, CD184 (CXCR4), CD185 (CXCR5), CD210 (IL‐10 receptor), CD267 (TACI), CD268 (BAFF receptor), CD274 (PD‐L1), CD360 (IL‐21 receptor), FcRL4 (CD307d), HLA‐DR, IgA, IgD, IgG_1_, IgG_2_, IgG_3_, IgG_4_ and IgM (Supplementary table 1). Markers not expressed on B cells (CD3) and CD45 (used as a barcode) were not included. TIM‐1 was used either as a surface or as an intracellular marker, so was not used in either algorithm. Conventional and more defined subsets of B cells were gated manually in FlowJo, using markers shown in Supplementary table 1.

Heatmaps were generated using pheatmap^45^ as part of the Spectre package^43^ in R. Each marker (column) was rescaled by minimum/maximum for each metacluster (row).

Manual gating was used to confirm changes identified by clustering results.^46^ B cells were differentiated into conventional B‐cell subsets and further defined using markers in Supplementary table 1. The quantity of each subset was calculated as a proportion of total B cells. When patient cell counts were made available, the proportion data were used to calculate the absolute number of each B‐cell subset.

### Mass cytometry statistical analysis

All statistics were calculated using packages available within R,^44^ using Type III Sum of Squares for PERMANOVA. sPLS‐DA was performed using the “mixOmics” package in R^47^ for feature selection of metaclusters that differ between groups of interest. A principal component analysis reduces the number of dimensions by summarizing the overall variance of a data set. The group that each individual belongs to does not affect the principal component analysis calculation. By contrast, an sPLS‐DA dimensionality reduction summarizes the differences between groups and calculates the parameters that contribute most to these differences. The “sparse” in sPLS‐DA removes parameters that do not contribute to differences. For the sPLS‐DA construction, M‐fold validation was used. The Mahalanobis distance was calculated, and at least three components were generated for each set of comparisons during sPLS‐DA construction. To calculate differences between groups, a PERMANOVA was done using the R package “vegan.”^48^ Permutation tests are powerful nonparametric tests, as they assume neither normality of distribution nor homogeneity of variance, and only assume data are exchangeable.^49^ Permutation tests randomly resample data without replacement, which allows them to accommodate small sample sizes.^50^ The same metaclusters selected by sPLS‐DA were used for the PERMANOVA. Data were then scaled (to allow balanced comparisons between parameters), with the Euclidean distance being calculated between points. A total of 4999 permutations were performed to generate *P*‐values (for a total of 5000 tests), to provide power and confidence for α = 0.01.^51^ For pairwise comparisons the package “pairwiseAdonis” was used with Holm’s correction for multiple comparisons.^52^

For comparisons of B‐cell subset/cluster levels (either proportions or cell counts) between all five groups [*non‐MS* controls, patients with untreated MS (*prior*), and patients with MS *post‐1*, *post‐2* alemtuzumab and *relapse*], a PERMANOVA was done followed by pairwise comparisons with Holm’s correction as discussed above. *Prior*, *post‐2* and *relapse* groups were compared with *non‐MS* controls (for three comparisons). When comparing between patients with MS before and after treatment, a linear mixed‐effects model was calculated using the “lme4” package.^53^ Individual patients were considered random effects for a repeated measures test that accommodated missing values, as not all patients had all available timepoints, while timepoints (*prior*, *post‐1*, *post‐2*) were fixed effects. A total of 4999 permutations were then run using the “permanova.lmer” function as part of the “predictmeans” R package to calculate *P*‐values.^54^, ^55^, ^56^ Five multiple comparisons were made: *prior* to *post‐1*, *post‐2* and *relapse*; and *post‐*1 to *post‐2* and *post‐2* to *relapse*. The functions “permmodels” and “predictmeans” (also part of the “predictmeans” package) generated 4999 permutations to calculate *P*‐values with Holm’s correction. A similar linear mixed‐effects model was used when comparing between median signal intensities of minor B‐cell subsets among nine *non‐MS* controls. Multiple comparisons with Holm’s correction were done when comparing between all minor subsets of the same major B‐cell population, for a total of 34 combinations.

For comparisons between patients with active or inactive disease, and patients with and without prior treatments, a permutation *t*‐test was done using the R package “RVAideMemoire”,^57^ using 4999 permutations.

All plots were generated using the R package “ggplot2”.^58^

### Single‐cell RNA sequencing data analysis

Single‐cell RNA sequencing data were obtained from Gene Expression Omnibus (accession number GSE133028).^11^ Data included single‐cell RNA sequencing data from PBMCs of healthy individuals and patients with RRMS (cohort GPL20301), and total RNA sequencing data from sorted B‐cell subsets (cohort GPL27644).

Single‐cell RNA sequencing data were processed as described by Ramesh *et al.,* ^11^ providing filtered single‐cell gene count matrices. Data were then log normalized using “NormalizeData” as part of the “Seurat” package in R.^59^ This cohort included PBMCs from healthy individuals (*n* = 3), and patients with neuromyelitis optica (*n* = 1), RRMS (*n* = 12), uveitis (*n* = 1) and clinically isolated syndrome (*n* = 2). All 175 895 cells underwent FlowSOM clustering and FIt‐SNE dimensionality reduction using the following genes to differentiate B cells from other major cell subsets (adapted from Ramesh *et al*.^11^): *APOE*, *C1QA*, *C1QB*, *C1QC*, *CCR7*, *CD3E*, *CD3D*, *CD4*, *CD8A*, *CD8B*, *CD14*, *CD19*, *CD40*, *CD40LG*, *CD79A*, *CD79B*, *CST3*, *FCER1A*, *FCGR3A*, *GNLY*, *HEMGN*, *IL7R*, *LILRA4*, *LYZ*, *MS4A1*, *MS4A7*, *NKG7*, *PPBP*, *TRAC*. FIt‐SNE plots were run with a “perplexity” of 1750 and “learning_rate” of 15 000, based on Kobak and Berens.^60^ B cells were identified and extracted for analyses. Only B cells from healthy controls and patients with RRMS were compared. Mean expression levels for each B cell were calculated and compared between healthy controls and patients with RRMS; 11/12 patients with RRMS were untreated, with 1/12 having previously received steroids. A permutation *t*‐test was done using the R package “RVAideMemoire,”^57^ using 4999 permutations. Genes chosen to compare were transcripts of the proteins analyzed in the mass cytometry panel. This included *PRDM1* (Blimp‐1), *BLNK*, *CD1D*, *CD5*, *MME* (CD10), *ITGAX* (CD11c), *CD19*, *MS4A1* (CD20), *CR2* (CD21), *FCER2* (CD23), *CD24*, *IL2RA* (CD25), *CD27*, *CD38*, *ENTPD1* (CD39), *CD40*, *TFRC* (CD71), *NT5E* (CD73), *CD79B*, *CD80*, *CD86*, *SDC1* (CD138), *CXCR4* (CD184), *CXCR5* (CD185), *IL10RA* (CD210), *TNFRSF13B* (CD267), *TNFRSF13C* (CD268), *CD274*, *IL21R* (CD360), *FCRL4*, *HLADRA*, *HLADRB1*, *HLADRB5*, *IGHA1*, *IGHA2*, *IGHD*, *IGHG1*, *IGHG2*, *IGHG3*, *IGHG4*, *IGHGM*, *HAVCR1* (TIM‐1).

Total RNA sequencing data of B‐cell subsets were processed as described by Ramesh *et al.,* ^11^ providing reads per kilobase per million for each sample. PBMCs were taken from six patients with untreated RRMS. PBMCs were then flow cytometry sorted into five B‐cell subsets: naïve (IgD^+^CD27^−^), double negative (IgD^−^CD27^−^), unswitched memory (IgD^+^CD27^+^), switched memory (IgD^−^CD27^+^) and plasmablasts/plasma cells (IgD^−^CD27^hi^). Provided reads per kilobase per million were compared between groups using a permutation of linear mixed‐effects model and multiple comparisons with Holm’s correction (10 in total) as described above. Genes chosen for comparisons were the same as those above.

## CONFLICT OF INTEREST

This work was in part funded by Sanofi‐Genzyme.

## Author Contributions

**Felix Marsh‐Wakefield:** Conceptualization; data curation; formal analysis; investigation; methodology; validation; visualization; writing – original draft; writing – review and editing. **Pierre Juillard:** Data curation; methodology; writing – review and editing. **Thomas Ashhurst:** Formal analysis; writing – review and editing. **Annette Juillard:** Data curation; methodology; writing – review and editing. **Diana Shinko:** Data curation; methodology; writing – review and editing. **Givanna H Putri:** Formal analysis; writing – review and editing. **Mark N Read:** Formal analysis; writing – review and editing. **Helen McGuire:** Methodology; writing – review and editing. **Scott N Byrne:** Conceptualization; funding acquisition; project administration; supervision; writing – review and editing. **Simon Hawke:** Conceptualization; funding acquisition; project administration; supervision; writing – review and editing. **Georges Grau:** Conceptualization; funding acquisition; project administration; supervision; writing – review and editing.

## Data Availability

The data that support the findings of this study are available from Sanofi‐Genzyme. Restrictions apply to the availability of these data, which were used under license for this study. Data are available from the authors with the permission of Sanofi‐Genzyme.

## References

[1] Thompson SA , Jones JL , Cox AL , Compston DA , Coles AJ . B‐cell reconstitution and BAFF after alemtuzumab (Campath‐1H) treatment of multiple sclerosis. J Clin Immunol 2010; 30: 99–105. 19763798 10.1007/s10875-009-9327-3

[2] Baker D , Herrod SS , Alvarez‐Gonzalez C , Zalewski L , Albor C , Schmierer K . Both cladribine and alemtuzumab may effect MS via B‐cell depletion. Neurol Neuroimmunol Neuroinflamm 2017; 4: e360. 28626781 10.1212/NXI.0000000000000360 PMC5459792

[3] Ceronie B , Jacobs BM , Baker D , *et al*. Cladribine treatment of multiple sclerosis is associated with depletion of memory B cells. J Neurol 2018; 265: 1199–1209. 29550884 10.1007/s00415-018-8830-y PMC5937883

[4] Kim Y, Kim G, Shin HJ *, et al*. Restoration of regulatory B cell deficiency following alemtuzumab therapy in patients with relapsing multiple sclerosis. J Neuroinflammation 2018; 15: 300. 30373595 10.1186/s12974-018-1334-y PMC6206644

[5] Akgun K , Blankenburg J , Marggraf M , Haase R , Ziemssen T . Event‐driven Immunoprofiling predicts return of disease activity in Alemtuzumab‐treated multiple sclerosis. Front Immunol 2020; 11: 56. 32082320 10.3389/fimmu.2020.00056 PMC7005935

[6] Gilmore W , Lund BT , Li P , *et al*. Repopulation of T, B, and NK cells following alemtuzumab treatment in relapsing‐remitting multiple sclerosis. J Neuroinflammation 2020; 17: 189. 32539719 10.1186/s12974-020-01847-9 PMC7296935

[7] Hilger C , Riedhammer C , Orso E , Weissert R . Effects of Alemtuzumab on (auto)antigen‐specific immune responses. Front Immunol 2020; 11: 563645. 33133074 10.3389/fimmu.2020.563645 PMC7578345

[8] Baker D , Marta M , Pryce G , Giovannoni G , Schmierer K . Memory B cells are major targets for effective immunotherapy in relapsing multiple sclerosis. EBioMedicine 2017; 16: 41–50. 28161400 10.1016/j.ebiom.2017.01.042 PMC5474520

[9] Havrdova E , Arnold DL , Cohen JA , *et al*. Alemtuzumab CARE‐MS I 5‐year follow‐up: durable efficacy in the absence of continuous MS therapy. Neurology 2017; 89: 1107–1116. 28835401 10.1212/WNL.0000000000004313 PMC5595278

[10] Van Gassen S , Callebaut B , Van Helden MJ , *et al*. FlowSOM: using self‐organizing maps for visualization and interpretation of cytometry data. Cytometry A 2015; 87: 636–645. 25573116 10.1002/cyto.a.22625

[11] Ramesh A , Schubert RD , Greenfield AL , *et al*. A pathogenic and clonally expanded B cell transcriptome in active multiple sclerosis. Proc Natl Acad Sci USA 2020; 117: 22932–22943. 32859762 10.1073/pnas.2008523117 PMC7502747

[12] Rubin RL , Tang FL , Chan EK , Pollard KM , Tsay G , Tan EM . IgG subclasses of autoantibodies in systemic lupus erythematosus, Sjogren's syndrome, and drug‐induced autoimmunity. J Immunol 1986; 137: 2528–2534. 3760566

[13] Gharavi AE , Harris EN , Lockshin MD , Hughes GR , Elkon KB . IgG subclass and light chain distribution of anticardiolipin and anti‐DNA antibodies in systemic lupus erythematosus. Ann Rheum Dis 1988; 47: 286–290. 3129997 10.1136/ard.47.4.286 PMC1003508

[14] Losy J , Mehta PD , Wisniewski HM . Identification of IgG subclasses' oligoclonal bands in multiple sclerosis CSF. Acta Neurol Scand 1990; 82: 4–8. 2239136 10.1111/j.1600-0404.1990.tb01578.x

[15] Graner M , Pointon T , Manton S , *et al*. Oligoclonal IgG antibodies in multiple sclerosis target patient‐specific peptides. PloS One 2020; 15: e0228883. 32084151 10.1371/journal.pone.0228883 PMC7034880

[16] Greve B , Magnusson CG , Melms A , Weissert R . Immunoglobulin isotypes reveal a predominant role of type 1 immunity in multiple sclerosis. J Neuroimmunol 2001; 121: 120–125. 11730948 10.1016/s0165-5728(01)00436-2

[17] Mohn N , Pfeuffer S , Ruck T , *et al*. Alemtuzumab therapy changes immunoglobulin levels in peripheral blood and CSF. Neurol Neuroimmunol Neuroinflamm 2020; 7: e654. 31826986 10.1212/NXI.0000000000000654 PMC7007635

[18] Trend S , Jones AP , Cha L , *et al*. Higher serum immunoglobulin G3 levels may predict the development of multiple sclerosis in individuals with clinically isolated syndrome. Front Immunol 2018; 9: 1590. 30057580 10.3389/fimmu.2018.01590 PMC6053531

[19] Kawano Y , Noma T , Yata J . Regulation of human IgG subclass production by cytokines. IFN‐gamma and IL‐6 act antagonistically in the induction of human IgG1 but additively in the induction of IgG2. J Immunol 1994; 153: 4948–4958. 7963558

[20] Rojas OL , Pröbstel A‐K , Porfilio EA , *et al*. Recirculating intestinal IgA‐producing cells regulate Neuroinflammation via IL‐10. Cell 2019; 176: 610–624.e18. 30612739 10.1016/j.cell.2018.11.035 PMC6903689

[21] Fehres CM , van Uden NO , Yeremenko NG , *et al*. APRIL induces a novel subset of IgA^+^ regulatory B cells that suppress inflammation via expression of IL‐10 and PD‐L1. Front Immunol 2019; 10: 1368. 31258536 10.3389/fimmu.2019.01368 PMC6587076

[22] Leffler J , Trend S , Ward NC , *et al*. Circulating memory B cells in early multiple sclerosis exhibit increased IgA^+^ cells, globally decreased BAFF‐R expression and an EBV‐related IgM^+^ cell signature. Front Immunol 2022; 13: e812317. 10.3389/fimmu.2022.812317 PMC8888440 35250986

[23] Fu C , Turck CW , Kurosaki T , Chan AC . BLNK: a central linker protein in B cell activation. Immunity 1998; 9: 93–103. 9697839 10.1016/s1074-7613(00)80591-9

[24] Minegishi Y , Rohrer J , Coustan‐Smith E , *et al*. An essential role for BLNK in human B cell development. Science 1999; 286: 1954–1957. 10583958 10.1126/science.286.5446.1954

[25] Pappu R , Cheng AM , Li B , *et al*. Requirement for B cell linker protein (BLNK) in B cell development. Science 1999; 286: 1949–1954. 10583957 10.1126/science.286.5446.1949

[26] Turkoglu R , Yilmaz V , Ozdemir O , *et al*. Peripheral blood B cell subset ratios and expression levels of B cell‐associated genes are altered in benign multiple sclerosis. Mult Scler Relat Disord 2021; 52: 103019. 34020389 10.1016/j.msard.2021.103019

[27] Ishiai M , Sugawara H , Kurosaki M , Kurosaki T . Cutting edge: Association of Phospholipase C‐γ2 Src homology 2 domains with BLNK is critical for B cell antigen receptor signaling. J Immunol 1999; 163: 1746–1749. 10438904

[28] Tan JE , Wong SC , Gan SK , Xu S , Lam KP . The adaptor protein BLNK is required for b cell antigen receptor‐induced activation of nuclear factor‐kappa B and cell cycle entry and survival of B lymphocytes. J Biol Chem 2001; 276: 20055–20063. 11274146 10.1074/jbc.M010800200

[29] Jin G , Hamaguchi Y , Matsushita T , *et al*. B‐cell linker protein expression contributes to controlling allergic and autoimmune diseases by mediating IL‐10 production in regulatory B cells. J Allergy Clin Immunol 2013; 131: 1674–1682. 23534976 10.1016/j.jaci.2013.01.044

[30] Rousset F , Garcia E , Defrance T , *et al*. Interleukin 10 is a potent growth and differentiation factor for activated human B lymphocytes. Proc Natl Acad Sci USA 1992; 89: 1890–1893. 1371884 10.1073/pnas.89.5.1890 PMC48559

[31] Levy Y , Brouet JC . Interleukin‐10 prevents spontaneous death of germinal center B cells by induction of the bcl‐2 protein. J Clin Invest 1994; 93: 424–428. 8282815 10.1172/JCI116977 PMC293803

[32] Kok LF , Marsh‐Wakefield F , Marshall JE , Gillis C , Halliday GM , Byrne SN . B cells are required for sunlight protection of mice from a CNS‐targeted autoimmune attack. J Autoimmun 2016; 73: 10–23. 27289166 10.1016/j.jaut.2016.05.016

[33] Nova‐Lamperti E , Fanelli G , Becker PD , *et al*. IL‐10‐produced by human transitional B‐cells down‐regulates CD86 expression on B‐cells leading to inhibition of CD4^+^ T‐cell responses. Sci Rep 2016; 6: 20044. 26795594 10.1038/srep20044 PMC4726240

[34] Briere F , Servet‐Delprat C , Bridon JM , Saint‐Remy JM , Banchereau J . Human interleukin 10 induces naive surface immunoglobulin D^+^ (sIgD^+^) B cells to secrete IgG1 and IgG3. J Exp Med 1994; 179: 757–762. 8294883 10.1084/jem.179.2.757 PMC2191366

[35] Australia New Zealand Multiple Sclerosis Genetics Consortium . Genome‐wide association study identifies new multiple sclerosis susceptibility loci on chromosomes 12 and 20. Nat Genet 2009; 41: 824–828. 19525955 10.1038/ng.396

[36] Yoshizaki A , Miyagaki T , DiLillo DJ , *et al*. Regulatory B cells control T‐cell autoimmunity through IL‐21‐dependent cognate interactions. Nature 2012; 491: 264–268. 23064231 10.1038/nature11501 PMC3493692

[37] Duddy ME , Alter A , Bar‐Or A . Distinct profiles of human B cell effector cytokines: a role in immune regulation? J Immunol 2004; 172: 3422–3427. 15004141 10.4049/jimmunol.172.6.3422

[38] Duddy M , Niino M , Adatia F , *et al*. Distinct effector cytokine profiles of memory and naive human B cell subsets and implication in multiple sclerosis. J Immunol 2007; 178: 6092–6099. 17475834 10.4049/jimmunol.178.10.6092

[39] Okada Y , Ochi H , Fujii C , *et al*. Signaling via toll‐like receptor 4 and CD40 in B cells plays a regulatory role in the pathogenesis of multiple sclerosis through interleukin‐10 production. J Autoimmun 2018; 88: 103–113. 29146546 10.1016/j.jaut.2017.10.011

[40] Jerram A , Guy TV , Beutler L , *et al*. Effects of storage time and temperature on highly multiparametric flow analysis of peripheral blood samples; implications for clinical trial samples. Biosci Rep 2021; 41: BSR20203827. 33600563 10.1042/BSR20203827 PMC7921292

[41] Wagar LE . Live cell barcoding for efficient analysis of small samples by mass Cytometry. Methods Mol Biol 2019; 1989: 125–135. 31077103 10.1007/978-1-4939-9454-0_9

[42] Linderman GC , Rachh M , Hoskins JG , Steinerberger S , Kluger Y . Fast interpolation‐based t‐SNE for improved visualization of single‐cell RNA‐seq data. Nat Methods 2019; 16: 243–245. 30742040 10.1038/s41592-018-0308-4 PMC6402590

[43] Ashhurst TM , Marsh‐Wakefield F , Putri GH , *et al*. Integration, exploration, and analysis of high‐dimensional single‐cell cytometry data using spectre. Cytometry A 2022; 101: 237–253. 33840138 10.1002/cyto.a.24350

[44] R Core Team . R: A Language and Environment for Statistical Computing. Vienna, Austria: R Foundation for Statistical Computing; 2021. http://www.R‐project.org/

[45] Kolde R. pheatmap: Pretty Heatmaps. 2019.

[46] Marsh‐Wakefield FM , Mitchell AJ , Norton SE , *et al*. Making the most of high‐dimensional cytometry data. Immunol Cell Biol 2021; 99: 680–696. 33797774 10.1111/imcb.12456 PMC8453896

[47] Rohart F , Gautier B , Singh A , Le Cao KA . mixOmics: an R package for 'omics feature selection and multiple data integration. PLoS Comput Biol 2017; 13: e1005752. 29099853 10.1371/journal.pcbi.1005752 PMC5687754

[48] Oksanen J , Blanchet FG , Friendly M *, et al*. vegan: Community Ecology Package 2019.

[49] Anderson MJ . A new method for non‐parametric multivariate analysis of variance. Austral Ecol 2001; 26: 32–46.

[50] Potter DM . A permutation test for inference in logistic regression with small‐ and moderate‐sized data sets. Stat Med 2005; 24: 693–708. 15515134 10.1002/sim.1931

[51] BFJ M . Randomization, Bootstrap and Monte Carlo Methods in Biology. 2nd ed. London: Chapman & Hall; 1997. 10.1201/9781315273075

[52] Arbizu PM pairwiseAdonis: Pairwise Multilevel Comparison using Adonis 2017.

[53] Bates D , Mächler M , Bolker B , Walker S . Fitting linear mixed‐effects models Usinglme4. J Stat Softw 2015; 67: 1–48.

[54] Lee OE , Braun TM . Permutation tests for random effects in linear mixed models. Biometrics 2012; 68: 486–493. 21950470 10.1111/j.1541-0420.2011.01675.x PMC3883440

[55] Luo D , Ganesh S & Koolaard J predictmeans: Calculate Predicted Means for Linear Models 2020.

[56] DeRamus T , Faghiri A , Iraji A , *et al*. Modular and state‐relevant functional network connectivity in high‐frequency eyes open vs eyes closed resting fMRI data. J Neurosci Methods 2021; 358: 109202. 33951454 10.1016/j.jneumeth.2021.109202 PMC10187826

[57] Hervé M. RVAideMemoire: Testing and Plotting Procedures for Biostatistics. 2021.

[58] Wickham H . ggplot2: Elegant Graphics for Data Analysis. New York: Springer‐Verlag; 2016.

[59] Hao Y , Hao S , Andersen‐Nissen E , *et al*. Integrated analysis of multimodal single‐cell data. Cell 2021; 184: 3573–3587.e29. 34062119 10.1016/j.cell.2021.04.048 PMC8238499

[60] Kobak D , Berens P . The art of using t‐SNE for single‐cell transcriptomics. Nat Commun 2019; 10: 5416. 31780648 10.1038/s41467-019-13056-x PMC6882829

