# Supplementary material for: Peripheral B‐cell dysregulation is associated with relapse after long‐term quiescence in patients with multiple sclerosis
Source: Immunol Cell Biol. 2022 May 12;100(6):453–67. doi: 10.1111/imcb.12552 (PMC9322415; doi:10.1111/imcb.12552)
Supplement: Supplementary file 1 [file IMCB-100-453-s001.pdf]

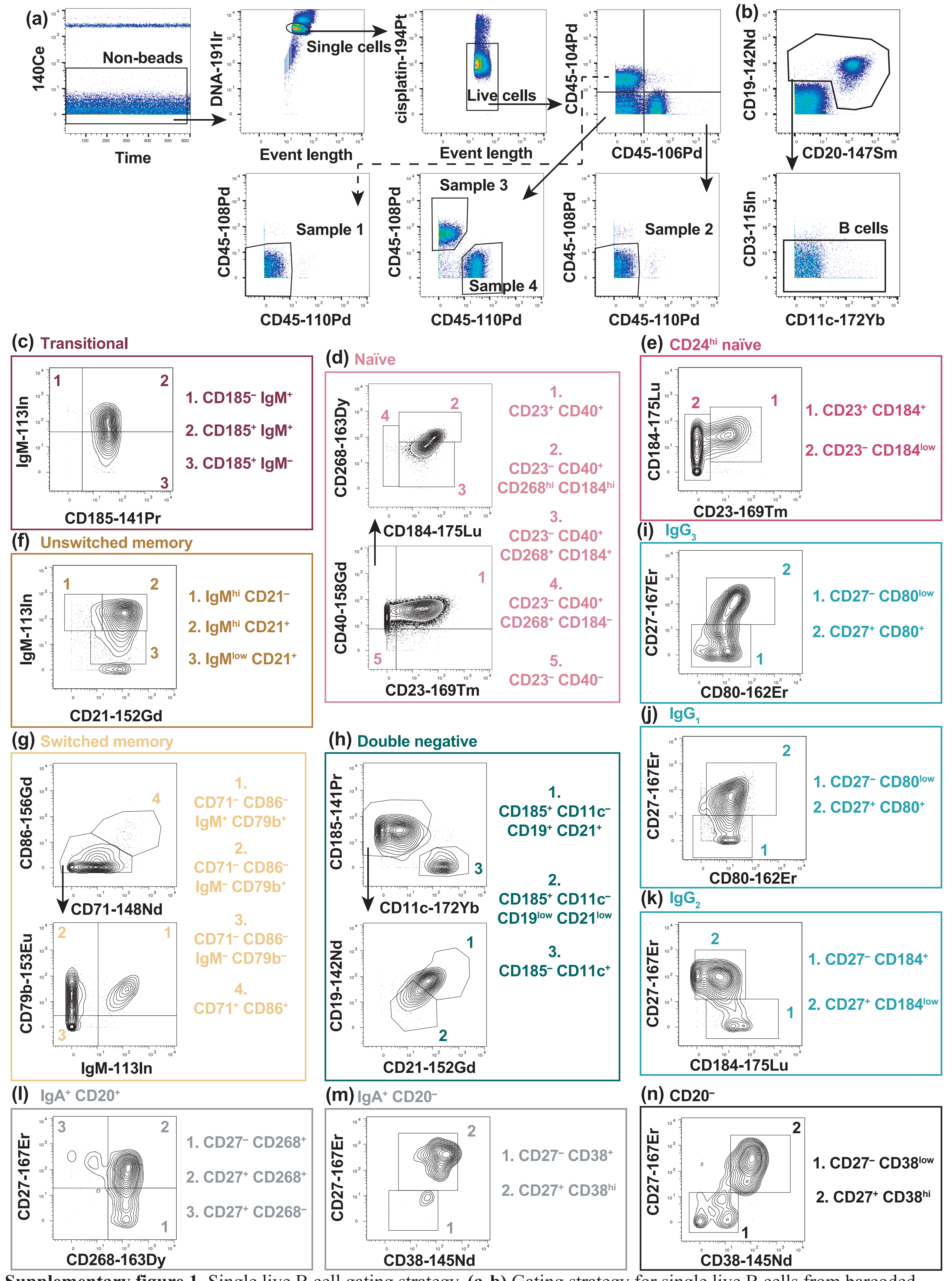


**Supplementary figure 1**. Single live B cell gating strategy. **(a-b)** Gating strategy for single live B cells from barcoded samples. Manual gating strategy of transitional **(c)**, naïve **(d)**, CD24$^{hi}$ naïve **(e)**, unswitched memory **(f)**, switched memory **(g)**, double negative **(h)**, IgG$_3$ **(i)**, IgG$_1$ **(j)**, IgG$_2$ **(k)**, IgA$^+$ CD20$^+$ **(l)**, IgA$^+$ CD20$^-$ **(m)** and CD20$^-$ **(n)** B cell subsets.

# Supplementary figure 2 page 1/4

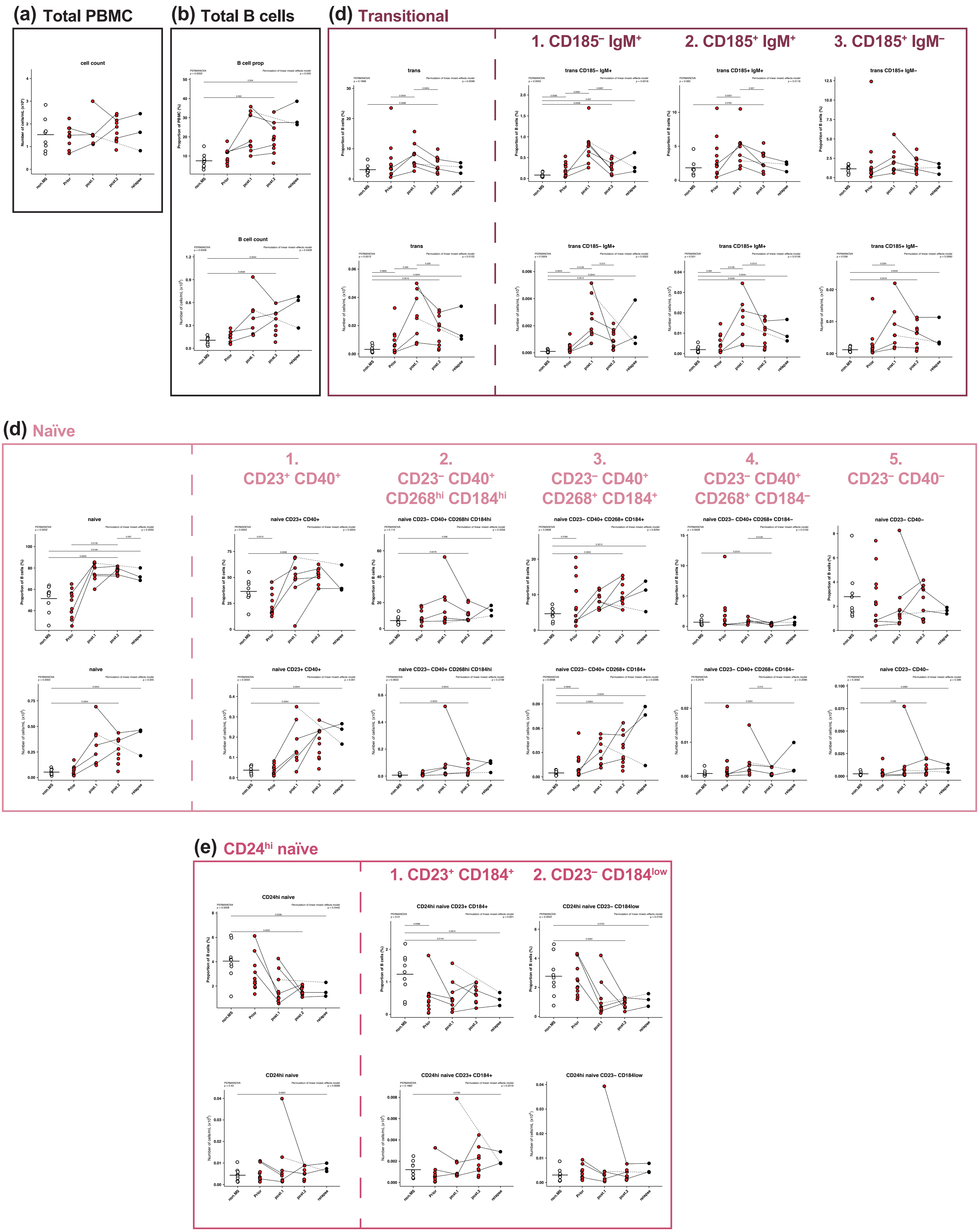

# Supplementary figure 2 page 2/4

## (f) Unswitched memory

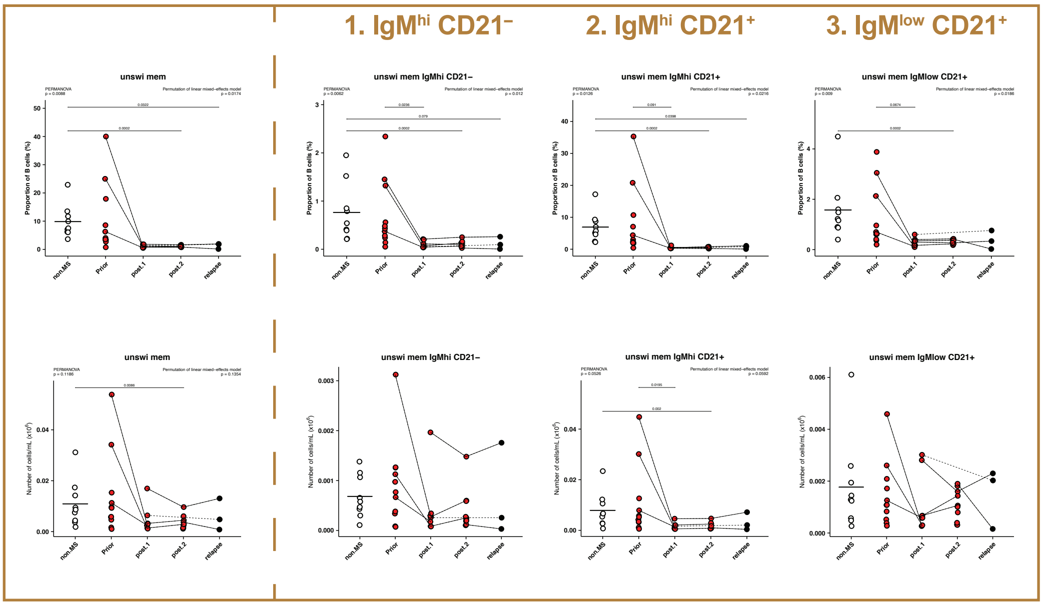


## (g) Switched memory

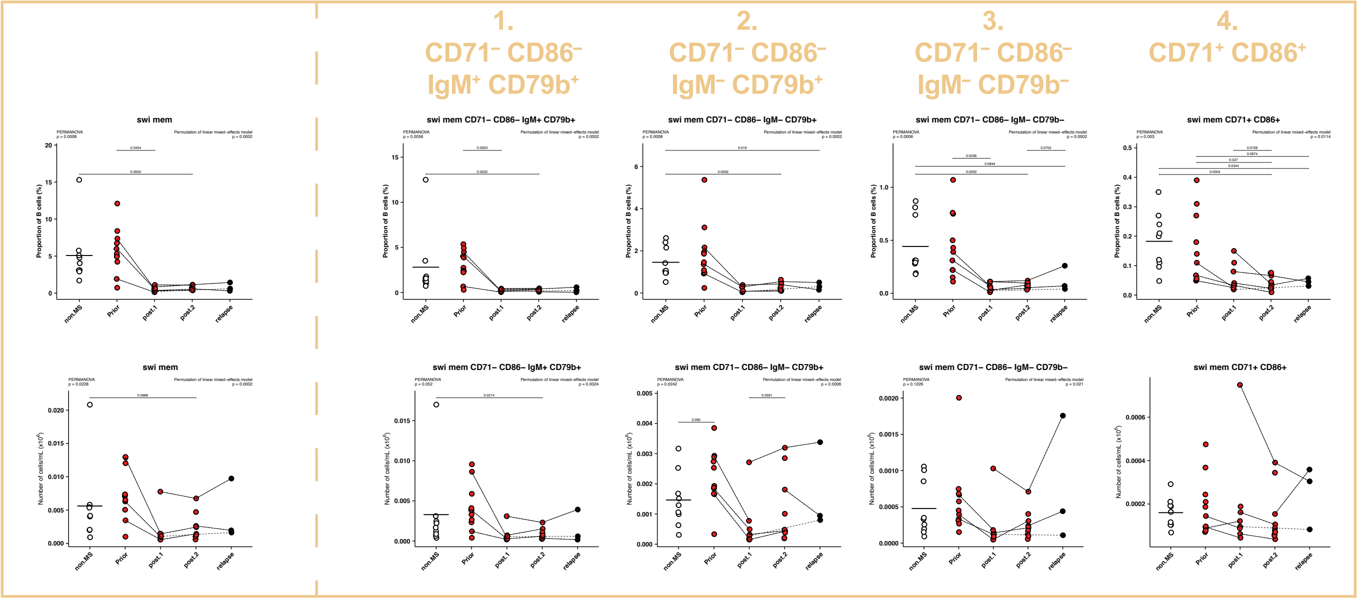


## (h) Double negative

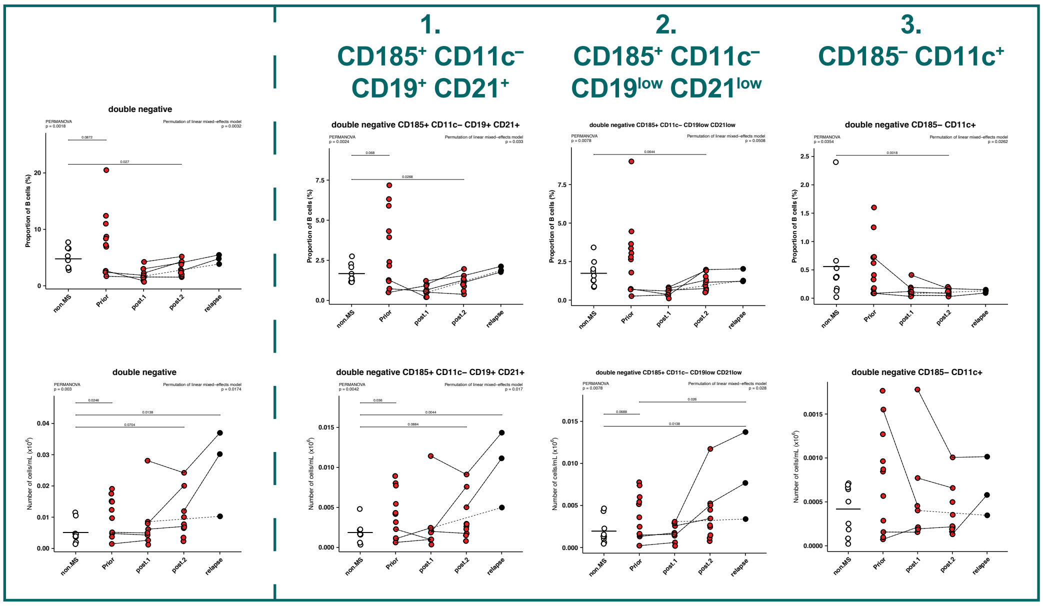

# Supplementary figure 2 page 3/4

**(i)** IgG$_3$

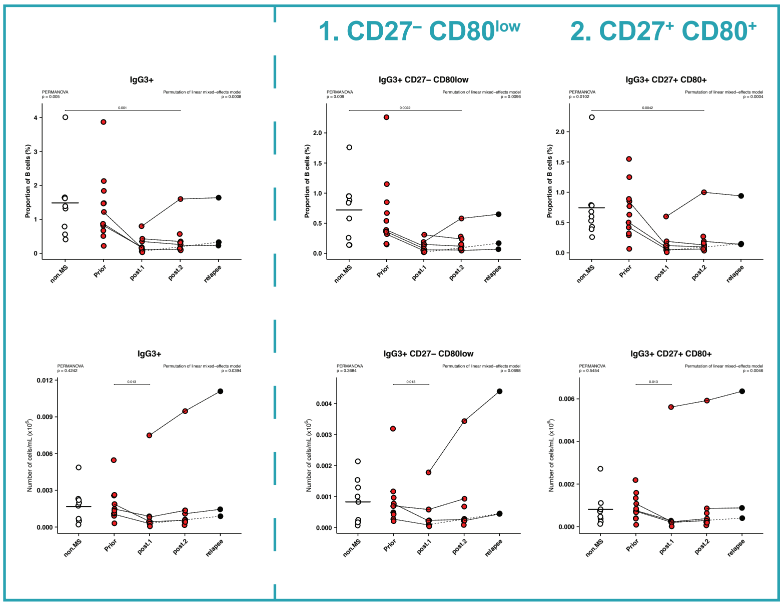


**(j)** IgG$_1$

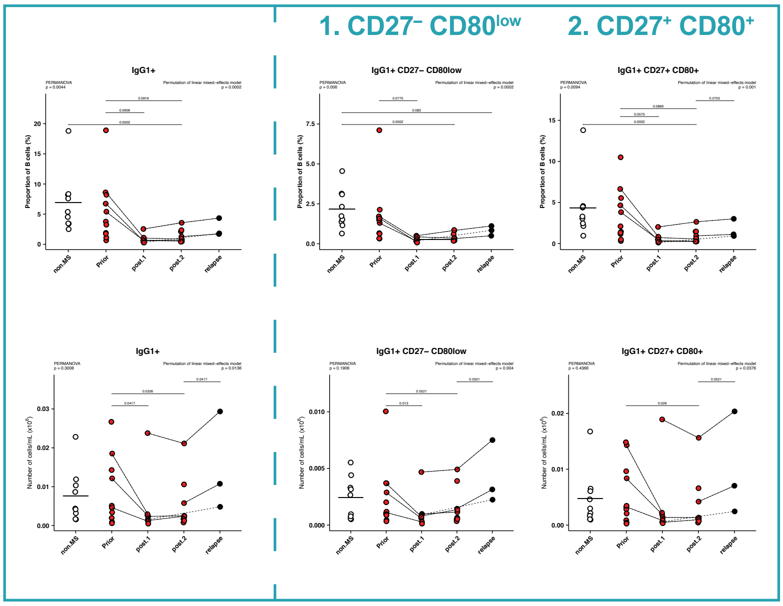


**(k)** IgG$_2$

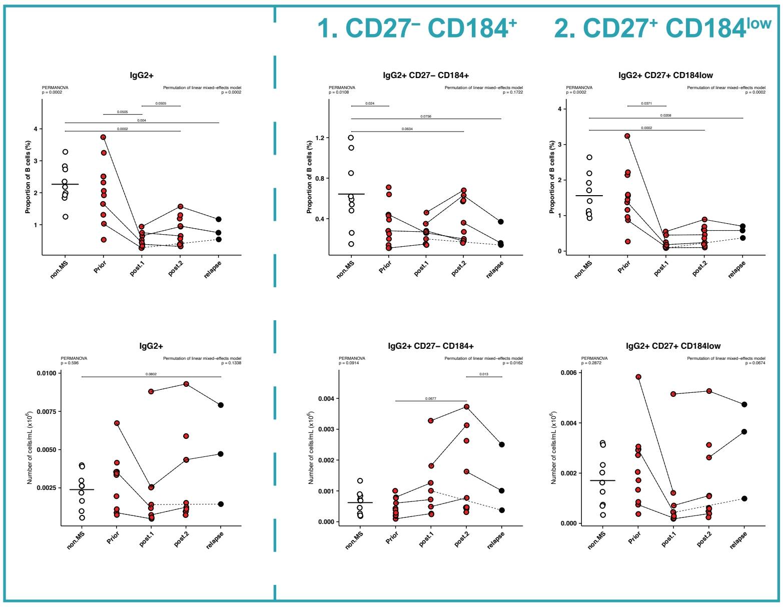


**(l)** IgG$_4$

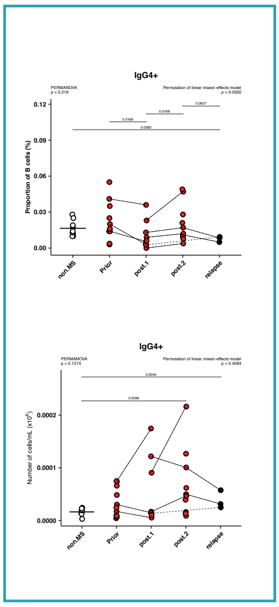

# Supplementary figure 2 page 4/4

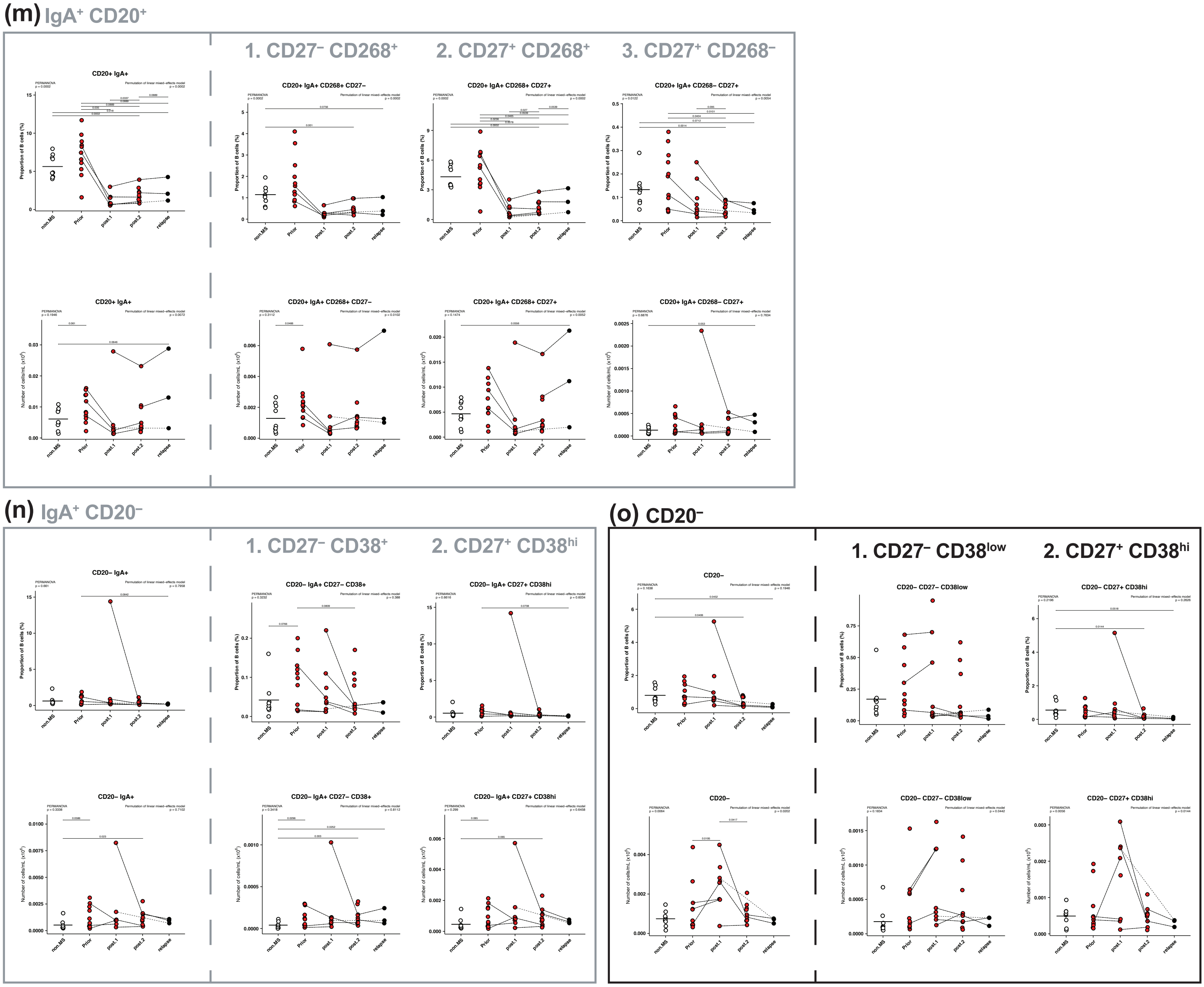


**Supplementary figure 2**. B cell subset levels across groups. **(a)** Total PBMC count across groups. Proportion and count data for total B cells **(b)**, transitional **(c)**, naïve **(d)**, CD24$^{hi}$ naïve **(e)**, unswitched memory **(f)**, switched memory **(g)**, double negative **(h)**, $IgG_3$ **(i)**, $IgG_1$ **(j)**, $IgG_2$ **(k)**, $IgG_4$ **(l)**, $IgA^+$ $CD20^+$ **(m)**, $IgA^+$ $CD20^-$ **(n)** and $CD20^-$ **(o)** B cell subsets. Solid lines signify data are available for adjacent timepoints, whilst dotted lines indicate patients with non-adjacent timepoints. For comparisons of B cell subset levels between all five groups (*non-MS* (multiple sclerosis) controls (n = 9), untreated MS patients (*prior*, n = 11), and MS patients *post-1* (up to twelve months after alemtuzumab dose, n = 9 proportion data and n = 8 count data), *post-2* (greater than twelve months, n = 10) alemtuzumab and *relapse* (n = 3)), a PERMANOVA was done followed pairwise comparisons with Holm's correction. *Prior*, *post-2* and *relapse* groups were compared to *non-MS* controls (for three comparisons). A linear mixed-effects model (LMM) was calculated when comparing between MS patients before and after treatment. 4999 permutations were then run to calculate *P*-values. Five multiple comparisons were made (*prior* to *post-1*, *post-2* and *relapse*, and *post-1* to *post-2*, and *post-2* to *relapse*) using a further 4999 permutations with Holm's correction. Mean is shown in *non-MS* controls, *P*-values < 0.1 are shown.

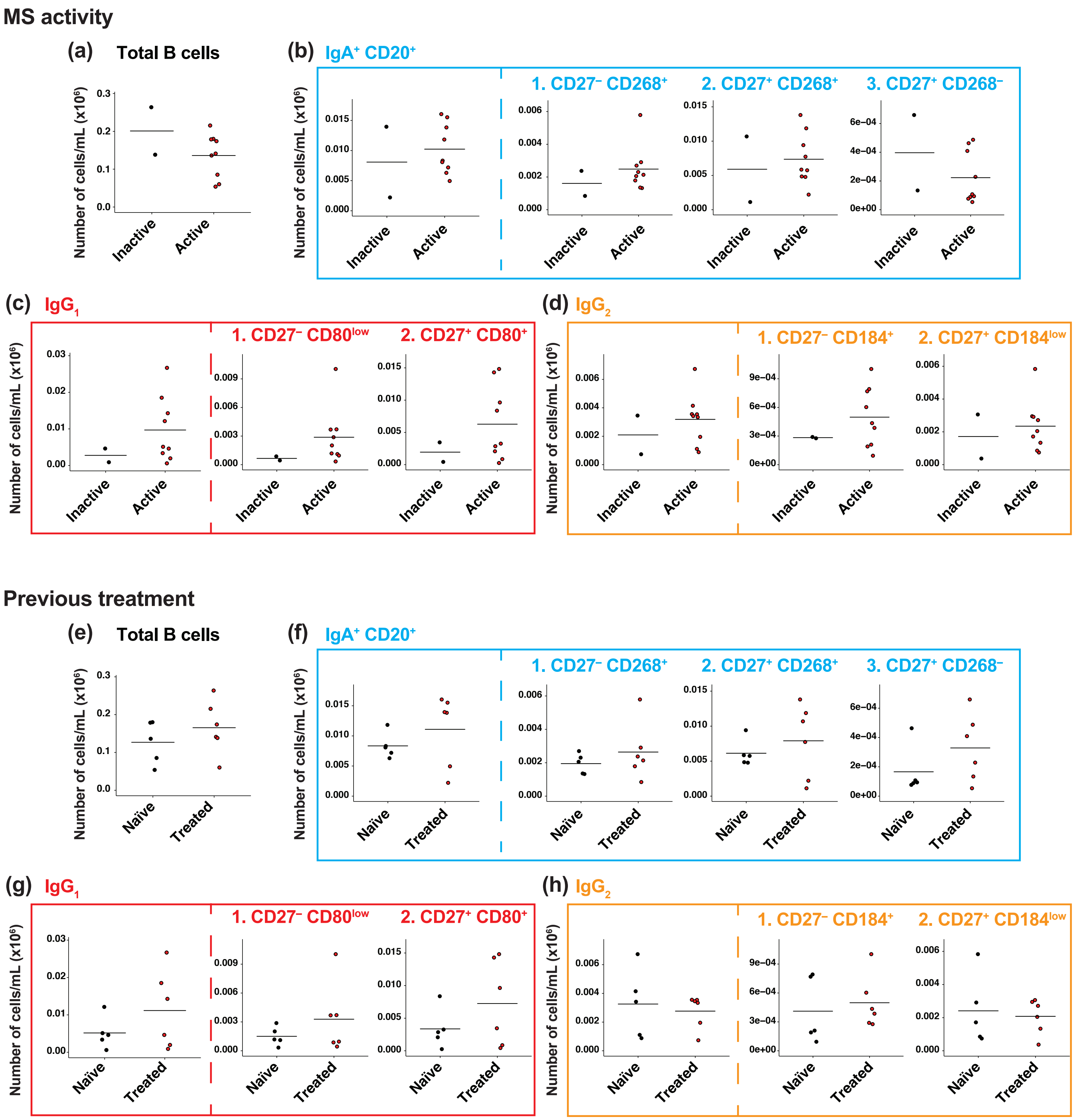


**Supplementary figure 3**. MS activity and prior treatments. **(a-d)** Comparions of inactive (n = 2) and active (n = 9) disease in multiple sclerosis (MS) patients prior to alemtuzumab treatment. **(e-h)** Comparions of untreated naïve (n = 5) and previously treated (n = 6) MS patients prior to alemtuzumab treatment. **(a,e)** total B cells, **(b,f)** IgA$^{+}$ CD20$^{+}$ B cell subsets, **(c,g)** $IgG_1$ B cell subsets, **(d,h)** $IgG_2$ B cell subsets. Permutation of *t*-test, mean shown.

# Supplementary figure 4 page 1/2

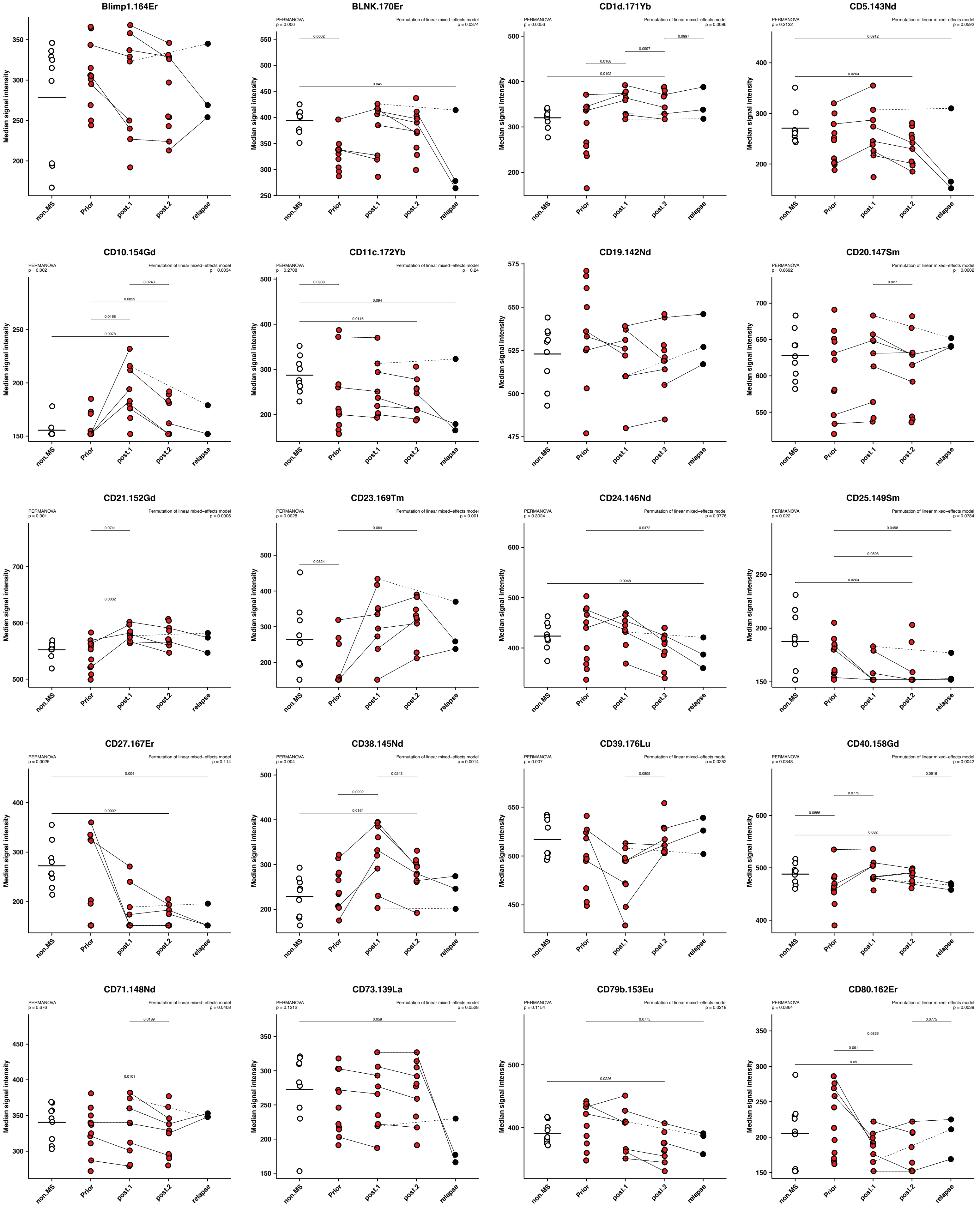

# Supplementary figure 4 page 2/2

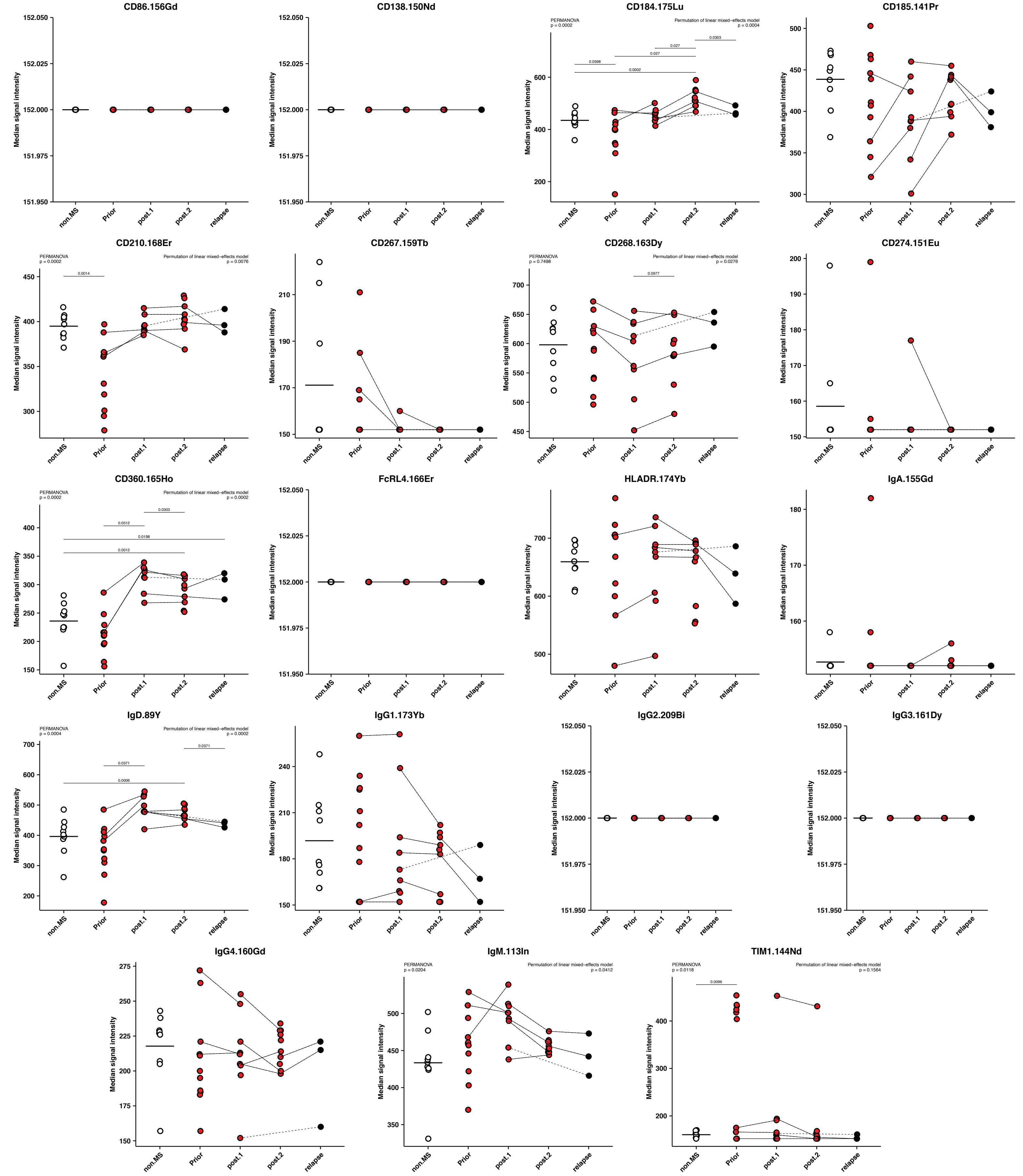


**Supplementary figure 4**. Median signal intensity of markers expressed by B cells across groups. Median signal intensity of marker expression by B cells across groups are shown. Solid lines signify data are available for adjacent timepoints, whilst dotted lines indicate patients with non-adjacent timepoints. For comparisons of B cell subset levels between all five groups (*non-MS* (multiple sclerosis) controls (n = 9), untreated MS patients (*prior*, n = 11), and MS patients *post-1* (up to twelve months after alemtuzumab dose, n = 9), *post-2* (greater than twelve months, n = 10) alemtuzumab and *relapse* (n = 3)), a PERMANOVA was done followed pairwise comparisons with Holm's correction. *Prior*, *post-2* and *relapse* groups were compared to *non-MS* controls (for three comparisons). A linear mixed-effects model (LMM) was calculated when comparing between MS patients before and after treatment. 4999 permutations were then run to calculate *P*-values. Five multiple comparisons were made (*prior* to *post-1*, *post-2* and *relapse*, and *post-1* to *post-2*, and *post-2* to *relapse*) using a further 4999 permutations with Holm's correction. Mean is shown in *non-MS* controls, *P*-values < 0.1 are shown.

Supplementary figure 5 page 1/7

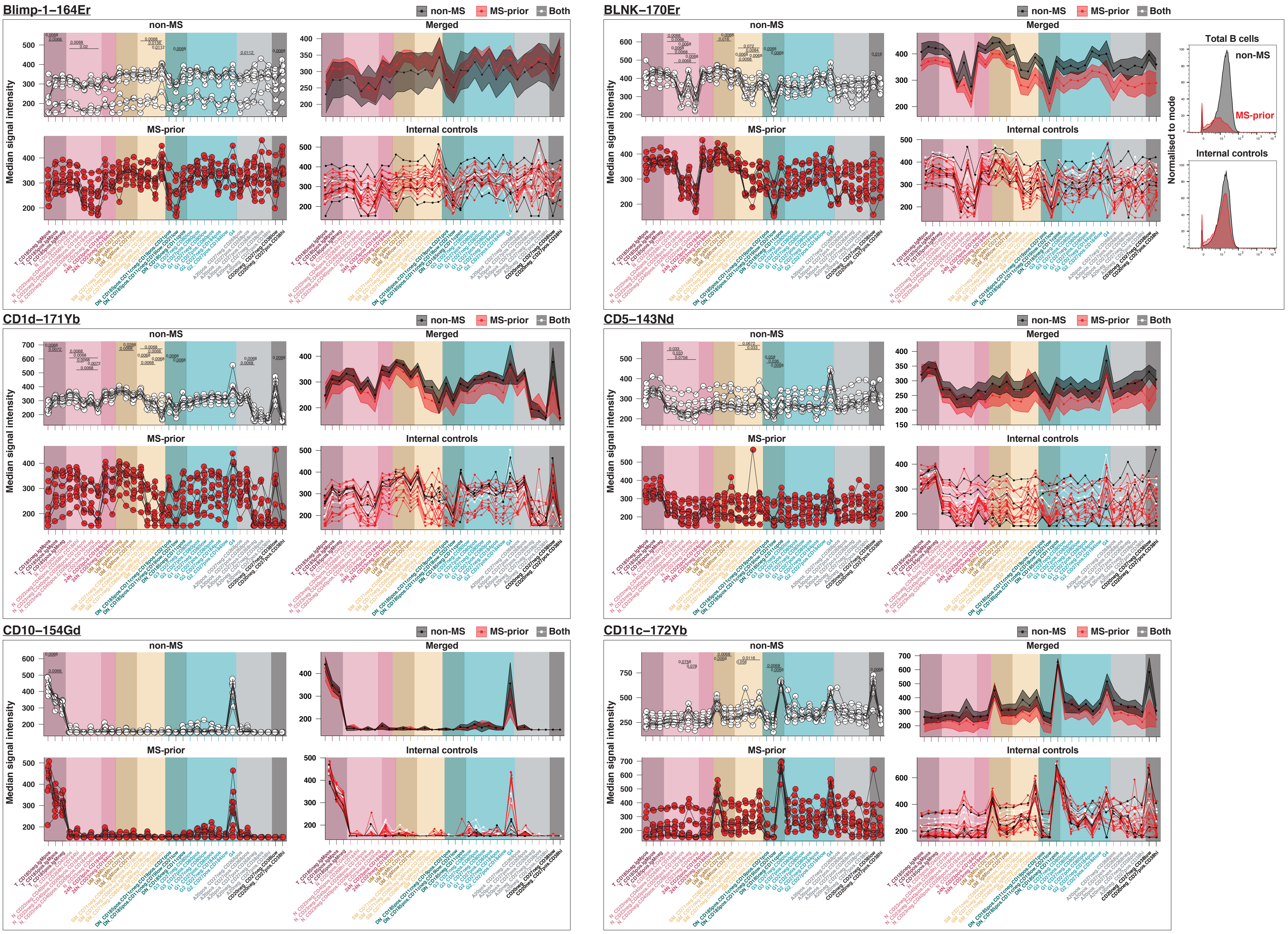

# Supplementary figure 5 page 2/7

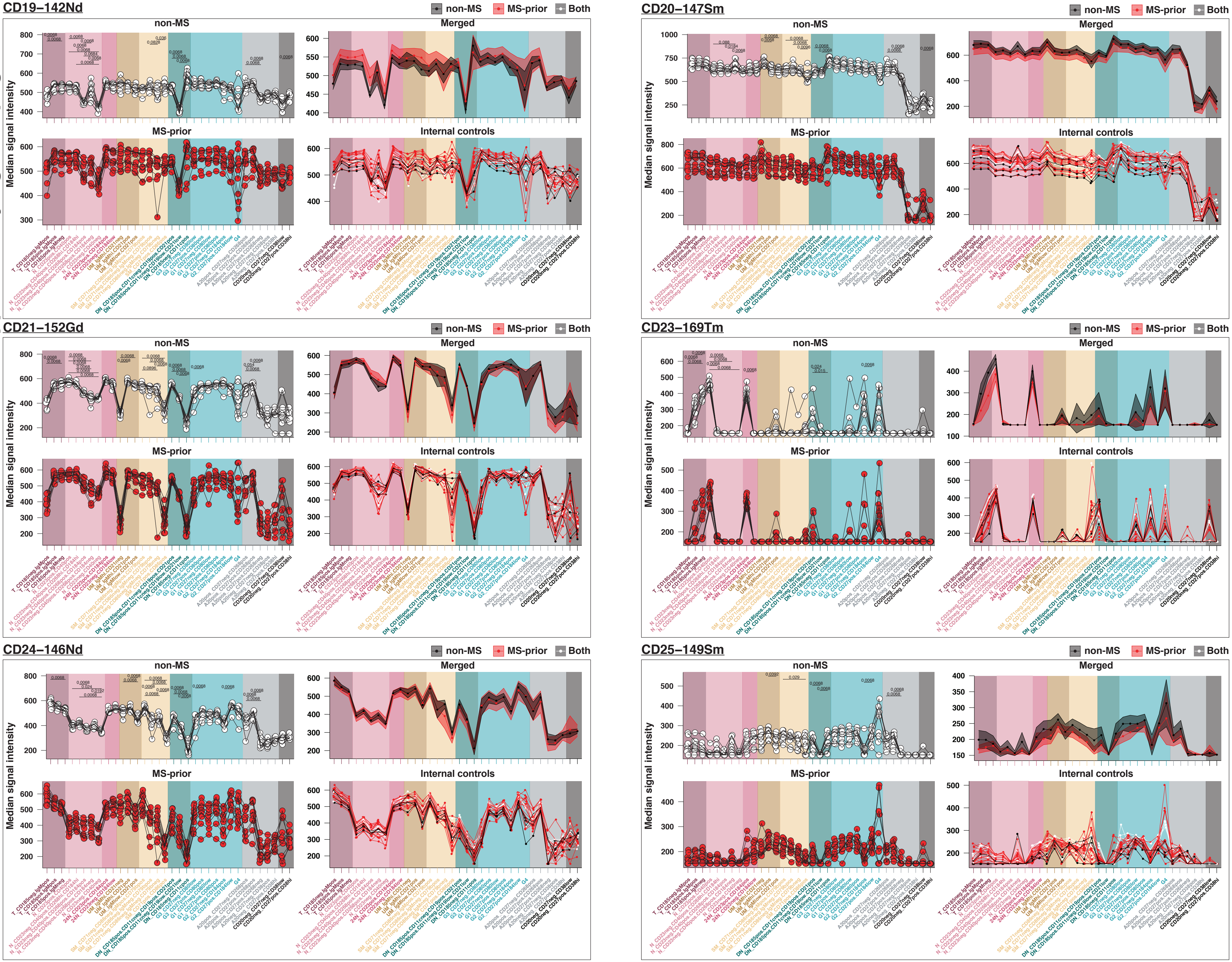

Supplementary figure 5 page 3/7

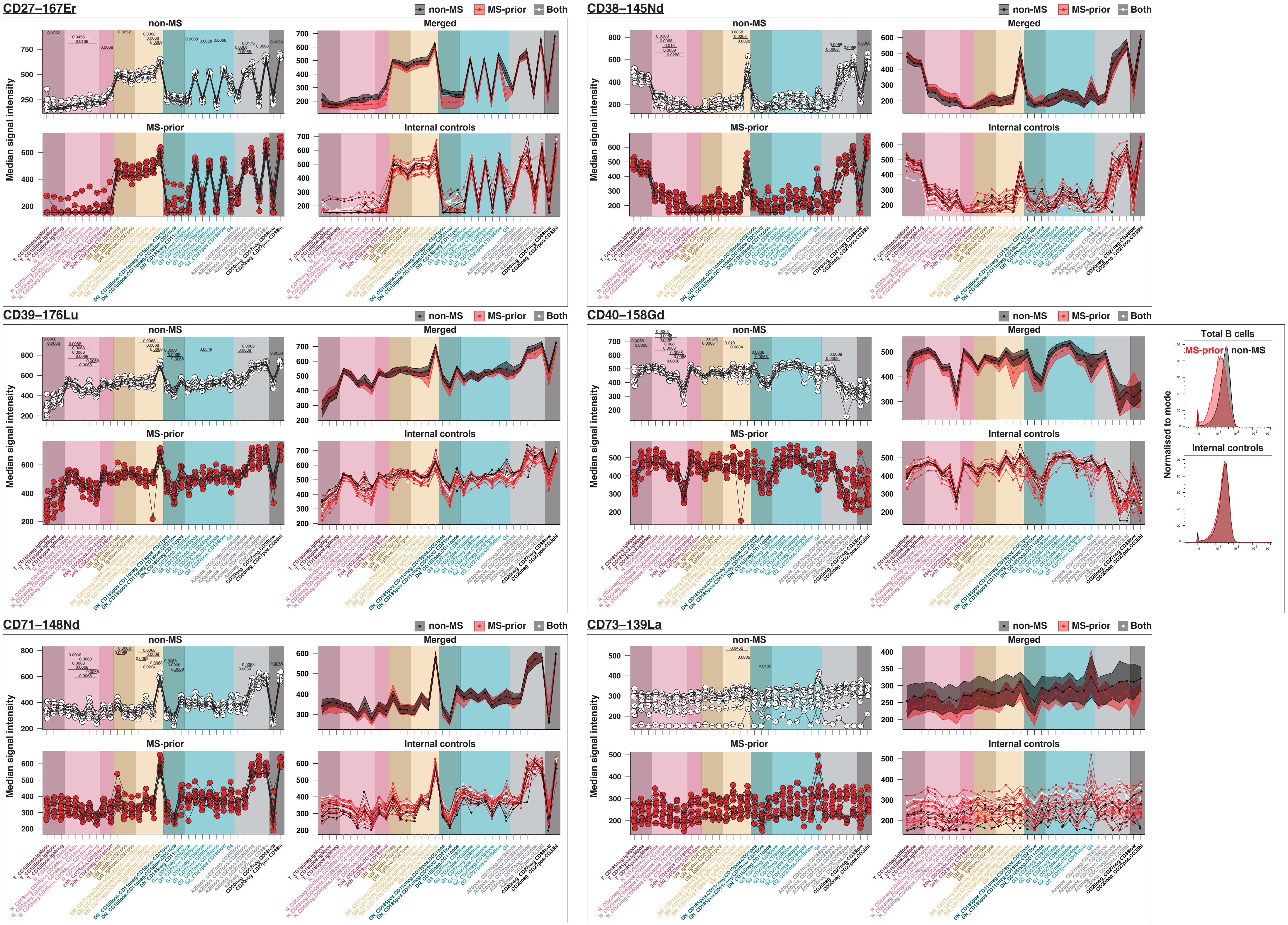

Supplementary figure 5 page 4/7

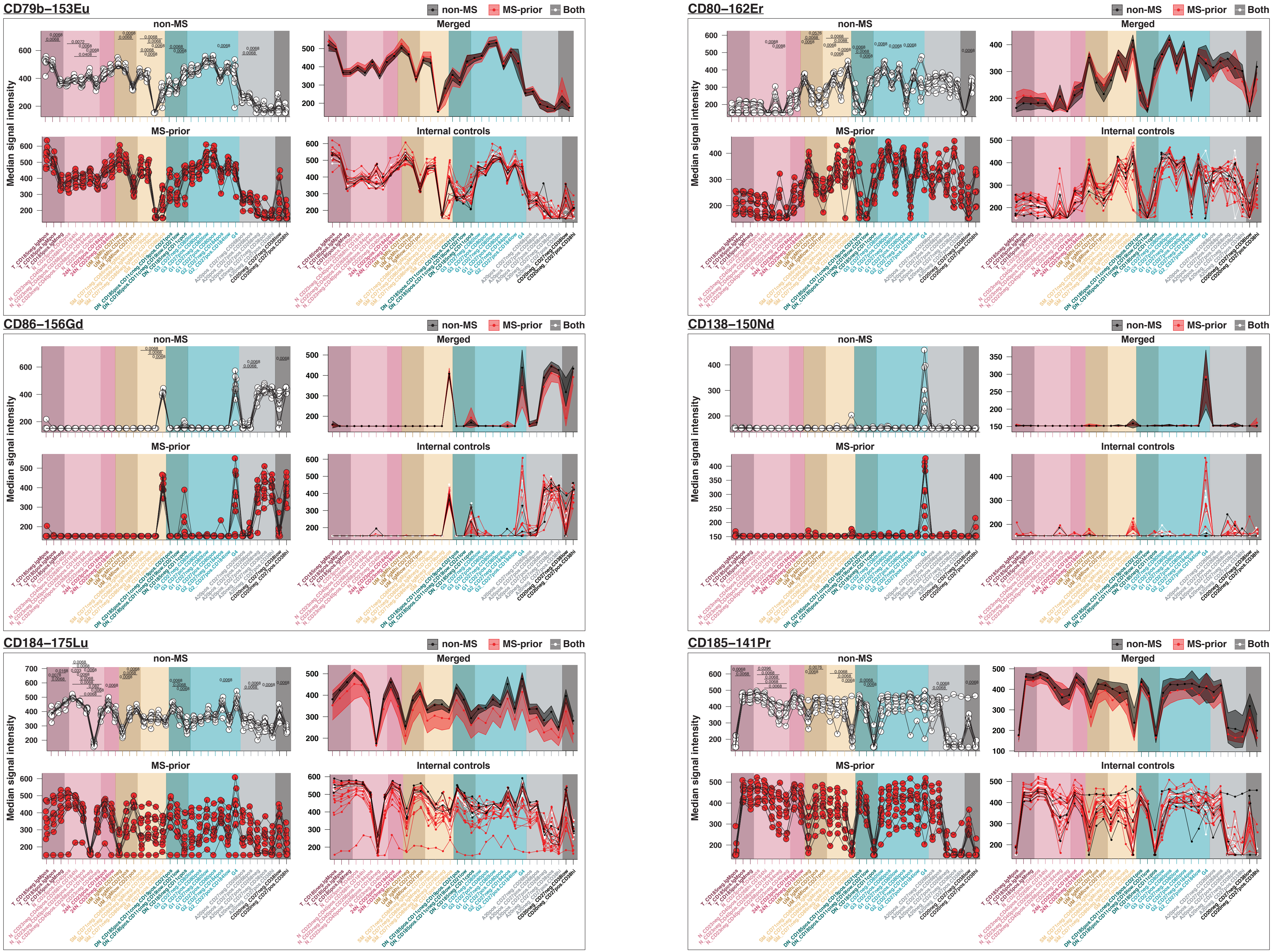

Supplementary figure 5 page 5/7

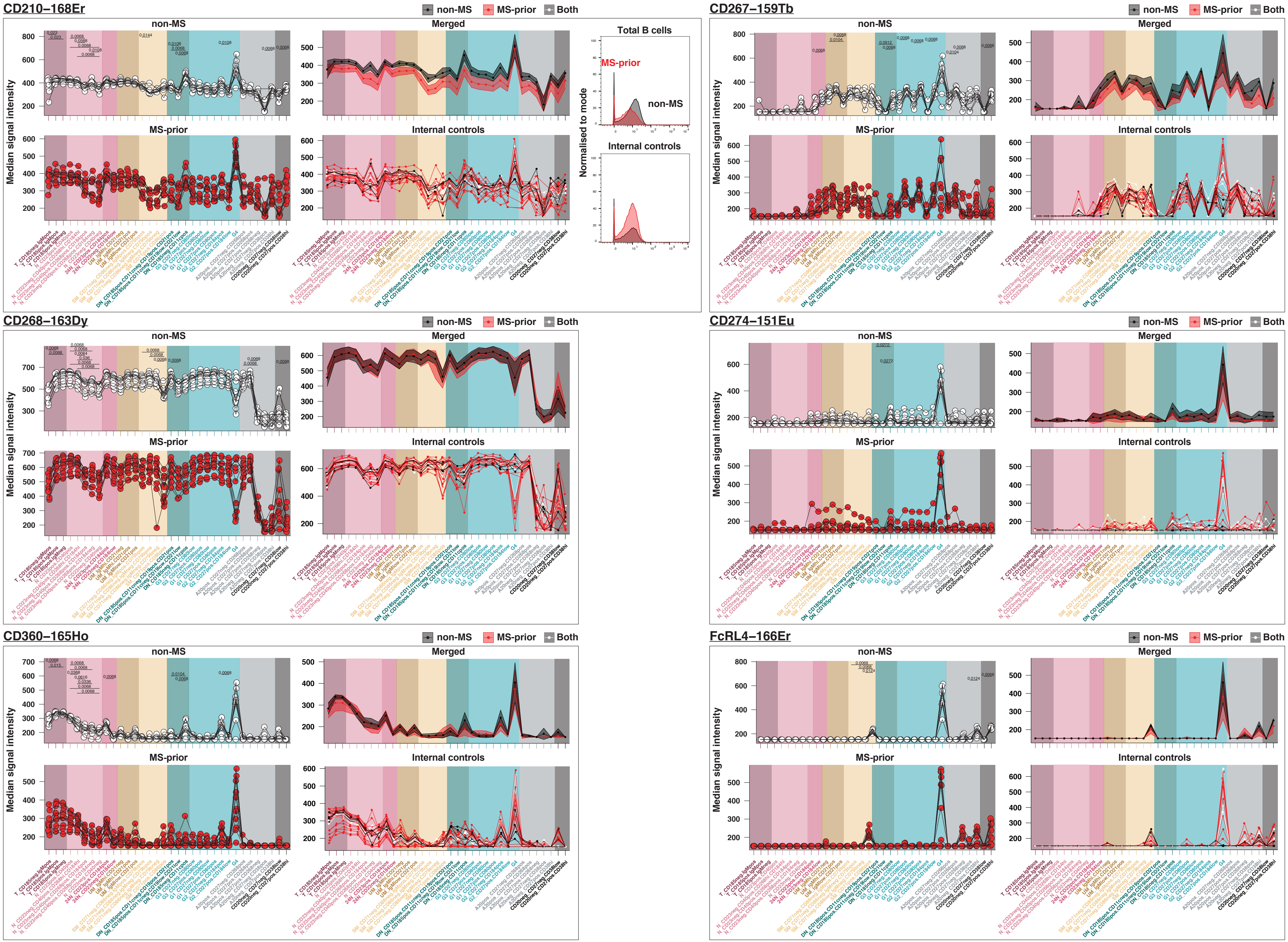

Supplementary figure 5 page 6/7

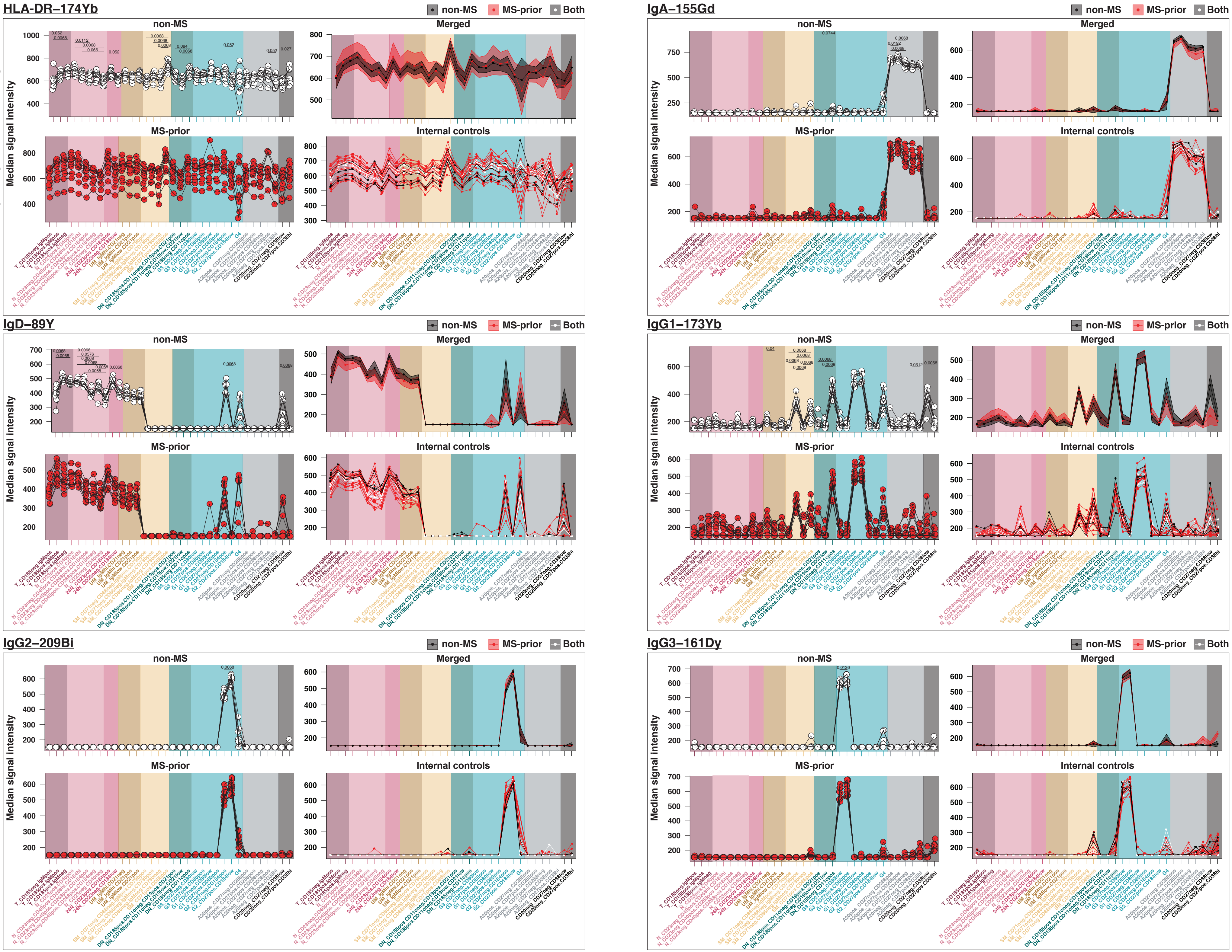

Supplementary figure 5 page 7/7

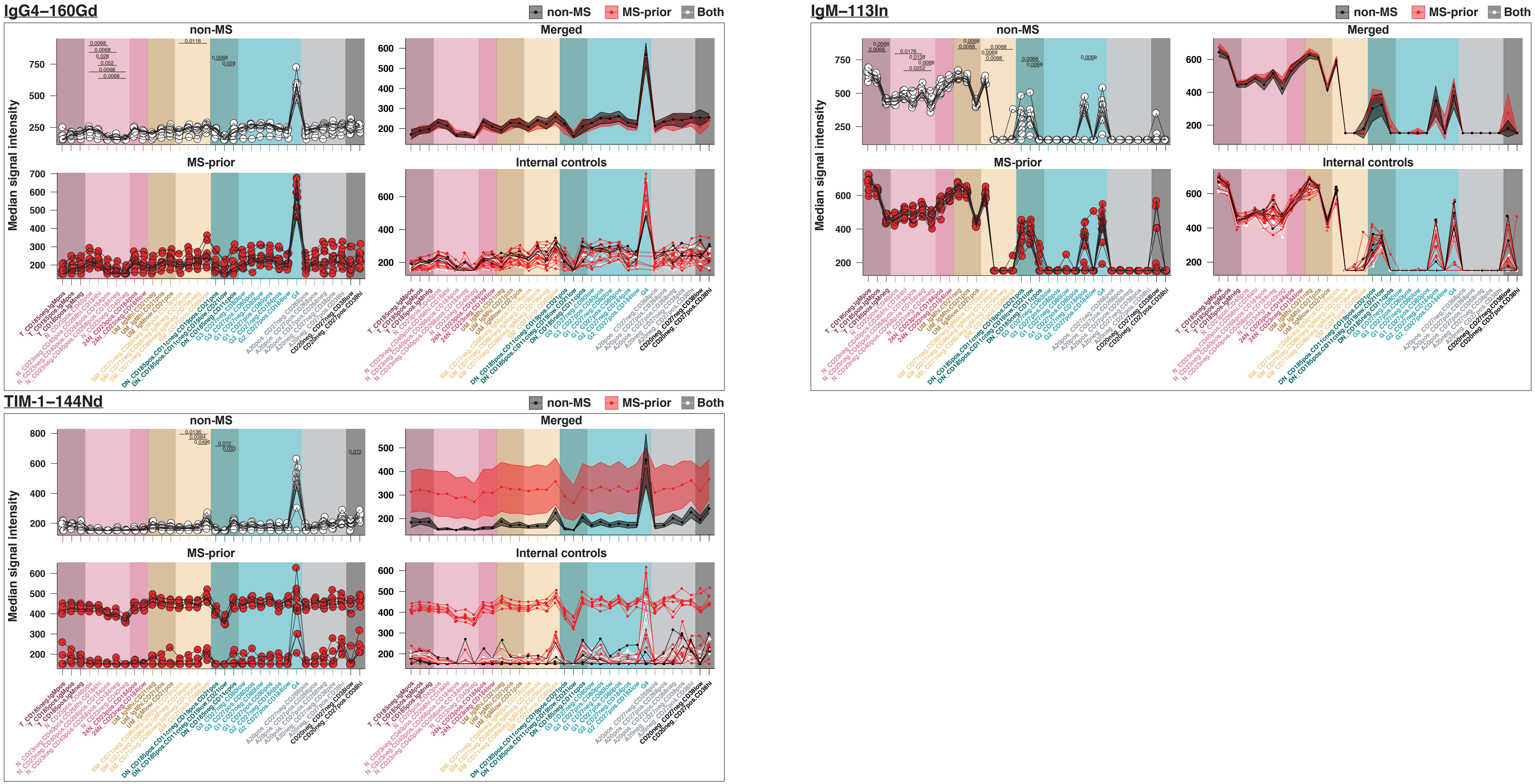


**TIM-1 was incorrectly used intracellularly at first which is why it’s so high in MS-prior**

**Supplementary figure 5**. Median signal intensity of markers across defined B cell subsets. The median signal intensity of each defined B cell subset was calculated for *non-MS* (multiple sclerosis, n = 9, white circles) and untreated MS patients (*prior*, n = 11, red circles). For the *non-MS* controls, a linear mixed-effects model was calculated with permutations (4999 run). Multiple comparisons were made between subsets of the same conventional B cell subset (34 comparisons in total) with 4999 permutations and Holm’s correction. Reported are *P*-values < 0.1. A combined plot was also generated, with mean and 95 % confidence interval shown for *non-MS* controls (black) and MS-*prior* (red). Internal controls from 25 batches, either alongside *non-MS* controls (black n = 4), MS-*prior* (red, n = 17) or a combination of both (white, n = 4) are shown to represent batch variability. BLNK, CD40 and CD210 have representative density plots for their expression between a *non-MS* control and MS-*prior*, and their internal controls. *T* transitional B cells, *N* naïve B cells, *24N* CD24$^{hi}$ B cells, *UM* unswitched memory B cells, *SM* switched memory B cells, *DN* double negative B cells, *G3* $IgG_3^+$ B cells, *G1* $IgG_1^+$ B cells, *G2* $IgG_2^+$ B cells, *G4* $IgG_4^+$ B cells, *A20+* $IgA^+$ $CD20^+$ B cells, *A20–* $IgA^+$ $CD20^-$ B cells, *CD20–* $CD20^-$ B cells.

**Supplementary Table 1**. Panel used for identifying B cells.

| Target | Isotope | Clone | Company | Algorithm calculations* | Conventional subset† | Defined subset‡ |
|---|---|---|---|---|---|---|
| Blimp-1# | 164Er§ | ROS195G | BioLegend | ✓ | | |
| BLNK# | 170Er | REA240 | Miltenyi Biotec | ✓ | | |
| CD1d | 171Yb | 51.1 | BioLegend | ✓ | | |
| CD3 | 115In | UCHT1 | BioLegend | | | |
| CD5 | 143Nd§ | UCHT2 | BioLegend | ✓ | | |
| CD10 | 154Sm§ | HI10a | BioLegend | ✓ | | |
| CD11c | 172Yb | Bu15 | BioLegend | ✓ | | ✓ |
| CD19 | 142Nd§ | HIB19 | BD Biosciences | ✓ | | ✓ |
| CD20 | 147Sm§ | 2H7 | BioLegend | ✓ | ✓ | |
| CD21 | 152Sm§ | BU32 | BioLegend | ✓ | | ✓ |
| CD23 | 169Tm§ | EBVCS-5 | BioLegend | ✓ | | ✓ |
| CD24 | 146Nd§ | ML5 | BioLegend | ✓ | ✓ | |
| CD25 | 149Sm§ | 2A3 | BioLegend | ✓ | | |
| CD27 | 167Er§ | M-T271 | BD Biosciences | ✓ | ✓ | ✓ |
| CD38 | 145Nd | HIT2 | BioLegend | ✓ | ✓ | ✓ |
| CD39 | 176Yb | A1 | BioLegend | ✓ | | |
| CD40 | 158Gd | 5C3 | BioLegend | ✓ | | ✓ |
| CD45¶ | 104Pd§ | HI30 | BioLegend | | | |
| | 106Pd§ | | BioLegend | | | |
| | 108Pd§ | | BioLegend | | | |
| | 110Pd§ | | BioLegend | | | |
| CD71 | 148Nd§ | CY1G4 | BioLegend | ✓ | | ✓ |
| CD73 | 139La | AD2 | BioLegend | ✓ | | |
| CD79b | 153Eu | CB3-1 | BioLegend | ✓ | | ✓ |
| CD80 | 162Er§ | L307.4 | BD Biosciences | ✓ | | ✓ |
| CD86 | 156Gd§ | IT2.2 | BD Biosciences | ✓ | | ✓ |
| CD138 | 150Nd§ | DL-101 | BioLegend | ✓ | | |
| CD184 (CXCR4) | 175Lu§ | 12G5 | BD Biosciences | ✓ | | ✓ |
| CD185 (CXCR5) | 141Pr | J252D4 | BioLegend | ✓ | | ✓ |
| CD210 (IL-10-R) | 168Er | REA239 | Miltenyi Biotec | ✓ | | |
| CD267 (TACI) | 159Tb | 1A1 | BioLegend | ✓ | | |
| CD268 (BAFF-R) | 163Dy§ | 11C1 | BioLegend | ✓ | | ✓ |
| CD274 (PD-L1) | 151Eu | 29E.2A3 | BioLegend | ✓ | | |
| CD360 (IL-21-R) | 165Ho | REA233 | Miltenyi Biotec | ✓ | | |
| FcRL4 (CD307d) | 166Er | 413D12 | BioLegend | ✓ | | |
| HLA-DR | 174Yb§ | L243 | BioLegend | ✓ | | |
| IgA | 155Gd | IS11-8E10 | Miltenyi Biotec | ✓ | ✓ | |
| IgD | 89Y§ | IA6-2 | BD Biosciences | ✓ | ✓ | |
| IgG1 | 173Yb | 12G8G11 | BioLegend | ✓ | ✓ | |
| IgG2 | 209Bi | HP6002 | BioLegend | ✓ | ✓ | |
| IgG3 | 161Dy | HP6047 | BioLegend | ✓ | ✓ | |
| IgG4 | 160Gd | IGHG4/1345 | Novus Biologicals | ✓ | ✓ | |
| IgM | 113In | MHM-88 | BioLegend | ✓ | | ✓ |
| TIM-1 | 144Nd | REA384 | Miltenyi Biotec | | | |

*Markers used for algorithms (including clustering and dimensionality reduction)
†Markers used for conventional B cell subsets
‡Markers used for defined B cell subsets
§Isotopes conjugated by the Ramaciotti Facility for Human Systems Biology, The University of Sydney, Australia.
¶CD45 was used for barcoding to allow up to three samples to be stained together.
#Markers stained intracellularly
